# Focal exposure of limited lung volumes to high-dose irradiation down-regulated organ development-related functions and up-regulated the immune response in mouse pulmonary tissues

**DOI:** 10.1186/s12863-016-0338-9

**Published:** 2016-01-27

**Authors:** Bu-Yeo Kim, Hee Jin, Yoon-Jin Lee, Ga-Young Kang, Jaeho Cho, Yun-Sil Lee

**Affiliations:** Herbal Medicine Research Division, Korea Institute of Oriental Medicine, Daejeon, 305-811 Republic of Korea; Graduate School of Pharmaceutical Sciences, Ewha Womans University, Seoul, 120-750 Korea; Division of Radiation Effects, Korea Institute of Radiological & Medical Sciences, Seoul, 139-706 Korea; Department of Radiation Oncology, Severance Hospital, Yonsei University College of Medicine, Seoul, 120-752 Korea

**Keywords:** Stereotactic body radiotherapy, Focal radiation, Microarray, Organ development, Immune response

## Abstract

**Background:**

Despite the emergence of stereotactic body radiotherapy (SBRT) for treatment of medically inoperable early-stage non-small-cell lung cancer patients, the molecular effects of focal exposure of limited lung volumes to high-dose radiation have not been fully characterized. This study was designed to identify molecular changes induced by focal high-dose irradiation using a mouse model of SBRT.

**Results:**

Central areas of the mouse left lung were focally-irradiated (3 mm in diameter) with a single high-dose of radiation (90 Gy). Temporal changes in gene expression in the irradiated and non-irradiated neighboring lung regions were analyzed by microarray. For comparison, the long-term effect (12 months) of 20 Gy radiation on a diffuse region of lung was also measured. The majority of genes were down-regulated in the focally-irradiated lung areas at 2 to 3 weeks after irradiation. This pattern of gene expression was clearly different than gene expression in the diffuse region of lungs exposed to low-dose radiation. Ontological and pathway analyses indicated these down-regulated genes were mainly associated with organ development. Although the number was small, genes that were up-regulated after focal irradiation were associated with immune-related functions. The temporal patterns of gene expression and the associated biological functions were also similar in non-irradiated neighboring lung regions, although statistical significance was greatly reduced when compared with those from focally-irradiated areas of the lung. From network analysis of temporally regulated genes, we identified inter-related modules associated with diverse functions, including organ development and the immune response, in both the focally-irradiated regions and non-irradiated neighboring lung regions.

**Conclusions:**

Focal exposure of lung tissue to high-dose radiation induced expression of genes associated with organ development and the immune response. This pattern of gene expression was also observed in non-irradiated neighboring areas of lung tissue, indicating a global lung response to focal high-dose irradiation.

**Electronic supplementary material:**

The online version of this article (doi:10.1186/s12863-016-0338-9) contains supplementary material, which is available to authorized users.

## Background

Radiation therapy is a standard treatment for patients with non-small-cell lung cancer (NSCLC). Currently, stereotactic body radiotherapy (SBRT) is considered an alternative treatment option for medically inoperable early-staged NSCLC patients, in which a high-dose of radiation is delivered repeatedly to tumor targets with great precision over one to five treatments [1, 2]. However, in spite of increasing evidence regarding the efficacy and safety of SBRT, especially with early-staged NSCLC, more accumulation of clinical cases and sufficient follow-up evaluation are required to draw conclusions regarding treatment outcomes after SBRT.

The efficacy of radiotherapy for lung cancer is severely compromised by the frequent occurrence of side effects, such as radiation-induced lung pneumonitis and fibrosis, which typically develop 6–24 months post-irradiation [3]. However, the time of onset and severity of lung injury after radiotherapy depends on many factors, including the volume of irradiated parenchyma, dose of absorbed radiation, and number of fractions [1, 4]. The incidence of pneumonitis for lung cancer patients treated with SBRT has been demonstrated to be 5–21 %. Moreover, the internal target volume and mean doses of radiation-exposed lung are major predictors of a higher pneumonitis incidence after SBRT [5, 6].

Despite clinical evidence of lung injury due to radiation, the molecular mechanisms underlying the effect of radiation have not been clearly identified. Recent reports suggest that various immune cells, cytokines, and regulatory molecules are involved in tissue reorganization and immune response modulation that occur in radiation-induced lung injury [4, 7, 8]. In addition, extracellular matrix (ECM) remodeling is involved in radiation-induced lung fibrosis [4, 9, 10]. Because ECM and its associated proteins, such as matrix metalloproteinase, provide physical support to tissues and serve as a major cytokine reservoir, loss of ECM regulation disrupts cell-cell junctions and affects the structural integrity of cells [11]. However, since these pathological changes in the lung can also be controlled by a combination of multiple regulatory pathways, a more comprehensive approach that involves whole genome-level investigation is necessary to understand the lung injury induced by radiation at the molecular level.

Previously, we developed a mouse model simulating clinical SBRT and validated the induction of lung injury by high-dose radiation [12–14]. In the present study, we used this SBRT model to measure how gene expression is altered by exposure of two lung areas to high-dose radiation: a directly irradiated region and non-irradiated neighboring region. Through functional bioinformatic analysis of genes altered by focal radiation, we attempted to understand biological changes occurring in the process of lung injury at the molecular level. Ultimately, our goal was to characterize the molecular pathology of lung injury induced by high-dose irradiation.

## Methods

### Mouse irradiation

All studies involving mice were approved by the Yonsei University Medical School Animal Care and Use Committee and were carried out in strict accordance with the recommendations in the Guide for the Care and Use of Laboratory Animals of the Yonsei University Medical School (The Yonsei University Medical School Animal Institution were approved by the AAALAC International). Five adult (10-week-old) male C57BL/6 mice (45 males) were housed per cage in a temperature controlled room at 22 ± 2 °C with a relative humidity of 50 ± 10 % and a 12 h dark/light cycle. Food and water were provided *ad libitum* throughout the experiment. To mimic SBRT conditions by irradiating only a small volume, we selected a 3-mm collimator to administer a 90 Gy dose to the central area of the left lung. To mimic conventional irradiation conditions, we delivered a 20 Gy dose with a 7-mm collimator, which almost covered the entire left lung. Radiation was delivered with an X-RAD 320 (Precision, North Branford, CT, USA), equipped with a collimator system composed of 5-cm-thick copper to produce focal radiation beams. Detailed methods have been described previously [12, 13]. During irradiation, the mice were anesthetized with an intraperitoneally administered mixture of 30 mg/kg of Zoletil and 10 mg/kg of Rompun. In the mice that underwent 90 Gy irradiation, focal irradiated tissues and neighboring tissues were isolated separately. In the mice that underwent 20 Gy irradiation of a diffuse area, the whole left lung was used. Control lungs were isolated from the non-irradiated mice.

### Tissue collection and histological examination

On the appropriate day after 90 Gy irradiation, directly irradiated region (focally irradiated area) and remaining area (neighboring area) of left lung were isolated from 3 mice. In the case of 20 Gy irradiation, whole left lungs (irradiated lung) from 3 mice were used. The mouse lung tissues were fixed in phosphate buffered 4 % formalin, and hematoxylin and eosin (H&E) and Masson’s Trichrome staining were performed as previously described [14].

### Microarray experiment

Total RNA from the mouse lung tissues was prepared using the Easy-SpinTM total RNA extraction kit according to the manufacturer’s instructions (iNtRON Biotechnology, Seoul, Republic of Korea). Before performing the microarray experiment, the quality of the purified RNA was measured using the Agilent 2100 Bioanalyzer (Agilent Technologies, Santa Clara, CA, USA); only samples with an RNA integrity number (RIN) greater than 7.0 were included in the microarray analysis. RNAs from triplicate experiments at each time point were pooled to exclude experimental bias. Isolated total RNA was amplified and labeled using the Low RNA Input Linear Amplification kit PLUS (Agilent Technologies) and then hybridized to a microarray containing approximately 44,000 probes (~21,600 unique genes), in accordance with the manufacturer’s instructions (Agilent Mouse whole genome 44K, Agilent Technologies). The arrays were scanned using an Agilent DNA Microarray Scanner (Agilent Technologies). The dataset is available online at the Gene Expression Omnibus (http://www.ncbi.nlm.nih.gov/geo) under the ID number GSE60541.

### Microarray data analysis

The raw intensity of the probe signals from the microarray was extracted using Feature Extraction Software (Agilent Technologies). Only probes showing signal intensity greater than 1.4 times the local background were selected and then normalized using the quantile method [15]. The expression ratio of genes in the experimental samples was obtained by comparing them with genes in the control sample. After averaging intensities for duplicated spots, the changes in gene expression were hierarchically clustered using average linkage algorithm of the Cluster program and visualized using the TreeView program (http://www.eisenlab.org).

### Quantitative real-time PCR (qRT-PCR) and immunohistochemistry

Real-time PCR analysis was performed using a CFX 96 Real Time PCR System (BioRad, Hercules, CA, USA) and the SensiFastTM Sybr HI-Rox Mix (Bioline, Taunton, MA, USA). The real-time PCR cycling conditions were as follows: 95 °C for 10 min, followed by 40 cycles for 10 s at 95 °C, 30 s at 60 °C, and 45 s at 72 °C. The primer sequences used were shown in Additional file 1. The relative changes in gene expression were analyzed by the 2-ΔΔCt method from real time quantitative PCR amplification. The immunohistochemistry staining was performed by using the VECTASTAIN Elite ABC Kit (VECTOR LABORATORIES INC., Burlingame, CA, USA) following the protocols provided by the manufacturer. Briefly, lung sections were deparaffinized with xylene, rehydrated though graded alcohol, and washed with PBS. Lung tissue sections were then treated with antigen retrieval buffers (abcam, Cambridge, UK) and 0.3 % H_2_O_2_ for 15 min at room temperature to block endogenous peroxidase activity. The primary antibodies used in this study were: Anti-CDKN1A (Santa Cruz Biotechnology, Santa Cruz, CA, USA). Immunoreactive sites were visualized using DAB (3,3′-diaminobenzidine) reagent set (KPL, Gaithersburg, MD, USA). The sections were counterstained with hematoxylin, rinsed with tap water, dehydrated and mounted. Images of lung tissue sections were taken with a digital camera mounted on an Axioscope A1 microscope (Carl Zeiss, Oberkochen, Germany).

### Temporal gene expression

The short time series expression miner (STEM) program was used to identify temporal changes in gene expression [16]. Only genes showing greater than two-fold variation at least one time from zero point were selected and then clustered using STEM clustering algorithm [17] with default settings of other parameters. The statistical significance of the resultant temporal expression pattern was calculated based on hypergeometric distribution, and then corrected using a false discovery rate (FDR) after 1,000 random permutations.

### Gene Ontology (GO) analyses

The Functional Annotation Tool of the Database for Annotation, Visualization, and Integrated Discovery (DAVID) was used for GO enrichment analyses [18]. The *p*-value of each GO-term was calculated using a modified Fisher’s exact test and was adjusted using the Benjamini-Hochberg FDR procedure. Since GO terms are composed of hierarchical structure, it is necessary to remove redundant GO terms to obtain clear biological interpretation of enriched GO terms. Using REVIGO program, we eliminated redundant GO terms from initially enriched GO terms (FDR <0.05) and constructed a network structure composed of non-redundant subsets of GO terms in which the distance between GO terms was measured based on the semantic similarity using whole UniProt GO database [19].

### Pathway analyses

A pathway enrichment analysis was also performed using the Functional Annotation Tool of DAVID [17]. From an input list of differentially expressed genes, significantly enriched pathways were calculated using a modified Fisher’s exact test and statistically adjusted by FDR.

For a more systematic pathway analysis, we conducted a Signaling Pathway Impact Analysis (SPIA) [20], which uses expression ratios of differentially genes, as well as lists of differentially expressed genes, to calculate signaling pathway topology. Specifically, by random bootstrap iteration of 3,000, two statistical measurements, P_NDE_ and P_PERT_, representing the over‐representation of input genes in a pathway and the abnormal perturbation of a specific pathway, respectively, were calculated. Then, the global *p*-value (P_G_) and were calculated from P_NDE_ and P_PERT_ for selection of significant pathways. P_G_, representing pathway rank, was calculated from the combined probability of both P_NDE_ and P_PERT_. The pathway information used in the present study was obtained from the database of Kyoto Encyclopedia of Genes and Genomes (KEGG, http://www.genome.jp/kegg/).

### Pathway activity

Previously outlined analytical methods for the pathway primarily focused on identification of enriched pathways using a group of differentially expressed genes. Therefore, to account for the accumulative effect of small changes by all genes in a pathway from the KEGG database, we linearly combined the logarithmic value of the expression of all of the genes in each pathway. When the gene product acted as repressor, the weight was multiplied by -1 and then the activity values were normalized by dividing by the size of the pathway [21]. The statistical significance for the measured activity was estimated using a random permutation-based method (*n* = 1,000) [22]. For each permutation, sample label was randomly permuted and the random pathway activities were estimated. Finally, FDR was determined by comparing the original activity value with randomly permutated values. Only pathways with an FDR below 0.01 were included in the clustering analysis.

### Functional network

A functional interaction network among gene products was constructed using the Reactome FI network cytoscape plugin application (http://www.reactome.org/), which utilizes a database (2013 version) of protein-protein interactions, gene co-expression, protein domain interaction, GO annotations, and text-mined protein interactions [23]. We used differentially expressed genes with at least two-fold variation as input microarray data. By implementing the Markov Cluster Algorithm on filtered genes with Pearson correlation coefficients greater than 0.8 in the interaction database, functional modules were identified and their associations with GO terms were measured using the REVIGO application [19].

## Results

### Fibrotic changes in the lung tissues focally irradiated with high-dose radiation (90 Gy)

**Fig. 1:**
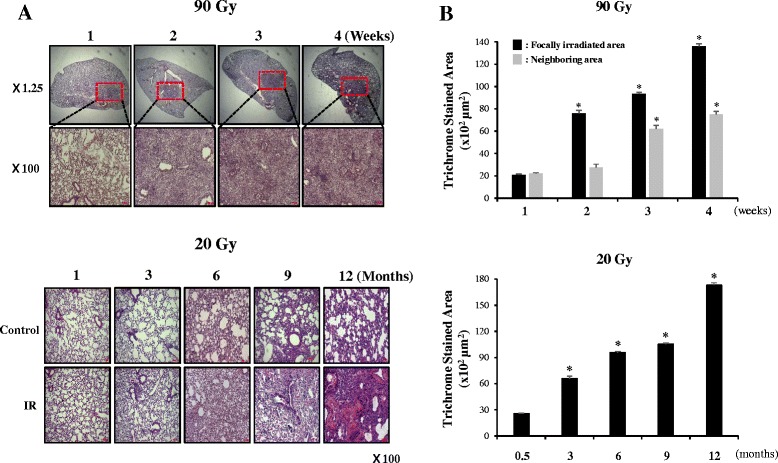
Fibrotic changes in the control or irradiated lung tissues after focal 90 Gy or 20 Gy irradiation. Mice were sacrificed at the indicated time points after irradiation, and the lungs were immersed in fixation solution. **a** Representative images of hematoxylin–eosin-stained lung sections from three mice are shown at each time point. The arrows indicate the focally irradiated area. **b** Lung sections were stained with Masson’s Trichrome stain to visualize blue-colored collagen deposition and quantitative assessments of the degree of collagen deposition were determined using an image J program (*p <0.05 *vs* non-irradiated control)

We carefully checked the animal status during the experiments and no death or apparent adverse effects were not detected. Histological changes were observed at each time point after focal exposure to high-dose radiation (90 Gy) in the central area of the left lungs of the mice. Significant abnormalities were observed subsequent to the focused, ablative dose radiation in the H&E-stained sections collected at different time points (Fig. 1a). Two weeks after irradiation, fibrotic changes were observed in the irradiated area, and at 4 weeks after irradiation, fibrous exudates were present in the air spaces. The detailed pathology of fibrosis has been described previously [12, 13]. Fibrotic changes were not observed in the tissues neighboring the focally irradiated areas of the lungs until 3 weeks. However, after 4 weeks, boundary regions of the focally irradiated areas became fibrotic. In the case of lungs that received 20 Gy, after 6 months, a significant amount of lung fibrosis was observed. To further confirm the fibrosis, lung sections were stained with Masson’s Trichrome to visualize the blue-colored collagen deposition. Quantitative graphs of the stained lung area are shown in Fig. 1b. No collagen was detected at 1 week after 90 Gy irradiation (data not shown). However, from the second week after irradiation, small amounts of collagen were detected and at 4 weeks, extensive collagen was observed, correlating with late-stage of fibrosis. Collagen deposition data for the areas neighboring the focally irradiated lung also showed a similar pattern of H&E staining. Collagen deposition was observed 3 weeks after irradiation. In the case of the lungs that received 20 Gy irradiation, a significant amount of lung fibrosis was observed 6 months after radiation, and peak collagen deposition was observed at 12 months after radiation. There were more fibrotic areas in the 90 Gy focally-irradiated lung regions than in the lungs that underwent 20 Gy whole lung irradiation.

### Gene expression profile in the lung tissue

**Fig. 2:**
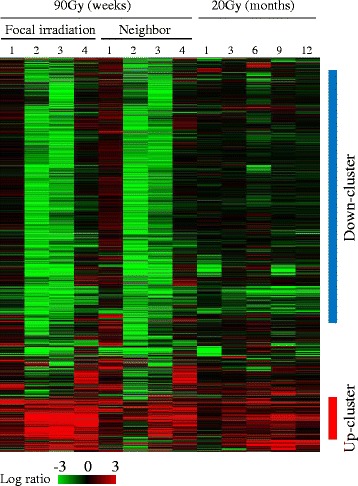
Temporal gene expression profile induced by radiation in mouse lung. Approximately 6,500 differentially expressed genes with a fold ratio greater than two or less than 0.5 (for up- and down-regulation, respectively) compared to the control in at least one sample were clustered hierarchically. Up-cluster and Down-cluster indicate the two sub-clusters. Columns and rows represent individual samples and genes, respectively. The expression ratio color scale ranges from *red* (high) to *green* (low), as indicated by the scale bar with log 2 units

Temporal changes of gene expression following focal lung exposure to high-dose (90 Gy) radiation and diffuse lung exposure to 20 Gy radiation were hierarchically clustered. As shown in Fig. 2, 1 week after focal irradiation with 90 Gy radiation, there were no noticeable changes in gene expression. But at 2 and 3 weeks after high-dose (90 Gy) irradiation, expression of a predominant number of genes were down-regulated and then restored to the control level at 4 weeks after irradiation (designated as “Down-cluster”). Interestingly this major pattern of gene expression was also observed in the non-irradiated neighboring lung areas but was not observed in the lungs irradiated with 20 Gy. In addition to the down-regulated pattern, we also identified another temporally up-regulated pattern of genes (designated as “Up-cluster”), especially in the focal area of lung received 90 Gy radiation.

**Fig. 3:**
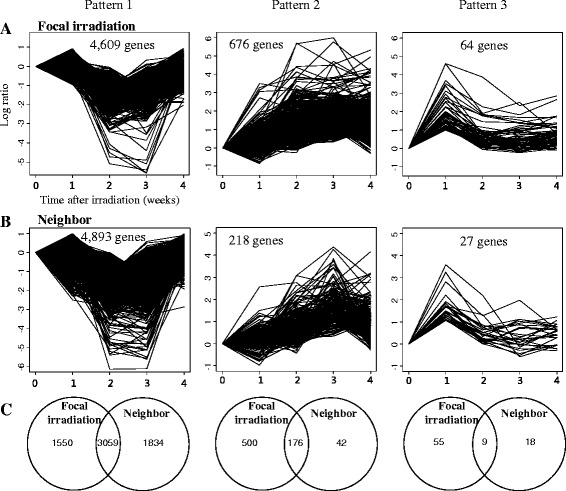
Temporal gene expression patterns by focal exposure to high-dose radiation. Temporally altered genes were identified by the Short Time-series Expression Miner (STEM) analysis in (**a**) focally-irradiated regions and (**b**) non-irradiated neighboring lung regions. Genes were classified into three patterns (FDR <0.001) in both lung regions. Pattern 1, Pattern 2, and Pattern 3 comprised 4609, 676, and 64 genes, respectively, in (**a**) focally irradiated regions, and 4893, 218, and 27, respectively, in (**b**) non-irradiated neighboring lung regions. (**c**) The number of common genes between the two lung regions was compared according to the three patterns

For more quantitative measurement of temporal changes of gene expression, time-series analysis was performed according to elapsed time after focally delivered high-dose radiation. Among diverse patterns of gene expression, we selected three patterns as representative (Fig. 3). “Pattern 1” was composed of down-regulated genes of 4609, “Pattern 2” was composed of up-regulated genes of 676, and final “Pattern 3” was composed of initially up-regulated genes of 64 from focally-irradiated regions (Fig. 3a). As also evidenced in Fig. 2, majority of genes among whole genes were included in Pattern 1, whereas Pattern 3 occupies only tiny part. However, considering that Pattern 1 shows delayed-response, initial response of Pattern 3 after focal irradiation could play a pivotal role in regulating overall expression of majority of genes. The gradual increase of gene expression in Pattern 2 could represents chronic response of lung induced by focal irradiation. Interestingly, these three patterns of gene regulation were also observed in non-irradiated neighboring lung regions in which 4893, 218, and 27 genes were presented as “Pattern 1”, “Pattern 2”, and “Pattern 3”, respectively (Fig. 3b). The two different areas of lung were compared in terms of common genes included in each pattern (Fig. 3c). Approximately 66.3 % (3059 of 4609 genes), 26.0 % (176 of 676 genes), and 14.0 % (9 of 64 genes) of Pattern 1, Pattern 2, and Pattern 3 genes, respectively, from the focally irradiated lung regions were also present in the non-irradiated neighboring lung regions. The full list of genes include in each pattern was shown in Additional file 2. Presence of different patterns of genes (FDR <0.01) implies the possible presence of different roles of genes acting on as a groups.

For the experimental confirmation of gene expression, some of key genes which are associated with many biological functions, were selected from each pattern and qRT-PCR experiments were performed on those genes. As shown in Additional file 1, genes from Pattern 1 were down-regulated, while genes from Pattern 2 were up-regulated in focally irradiated lung regions in accordance with microarray results of Fig. 3. We also confirmed that protein amount of CDKN1A, one of Pattern 3 gene, was correlated with gene expressional change using immunohistochemistry assay (Additional file 1).

Interestingly, we also observed two temporal patterns of gene expression (Down-pattern and Up-pattern) in lung tissue irradiated with 20 Gy. Although the number of genes in these patterns was smaller than those from high-dose (90 Gy) radiation, 53.7 % (79 of 147 genes) and 46.8 % (68 of 145 genes) were commonly present in Pattern 1 and Pattern 2 from directly-exposed regions of lung that received high-dose radiation (Additional file 3), implying the presence of commonly responding genes in the setting of different radiation doses.

### GO analysis

To identify the biological function associated with the three expressional patterns induced by focal exposure to high-dose (90 Gy) radiation, a GO analysis was performed. The most significant GO terms associated with the three patterns (FDR <0.01) are shown in Table 1 (for the full list of enriched GO terms, please see Additional file 4). Pattern 1 was mainly enriched with organ development-related GO terms, such as cell adhesion (GO:0016337), heart development (GO:0007507), and organ morphogenesis (GO:0048562 and GO: 0007389) (FDR <0.01). Another intriguing GO category enriched in Pattern 1 was a transcription regulation-related term (GO:0006355). In contrast, immune-related GO terms, including immune response (GO:0006955), defense response (GO:0006952), inflammatory response (GO:0006954), and wound response (GO:0009611), were enriched in Pattern 2 (FDR <0.01). Finally, cell cycle-related GO terms, such as cell cycle arrest (GO:0007050), cell cycle process (GO:0022402), and apoptosis (GO:0006915), were enriched in Pattern 3 (FDR <0.01).

**Table 1:** Top 10 GO terms significantly enriched (FDR < 0.01) in lung focally exposed to high-dose radiation of 90 Gy

Focally irradiated area											
Pattern 1				Pattern 2				Pattern 3			
GO ID	Name	*p*-value*	FDR^a^	ID	Name	*p*-value	FDR	ID	Name	*p*-value	FDR
GO:0016337	Cell-cell adhesion	7.33E-10	2.67E-06	GO:0006955	Immune response	2.64E-63	5.58E-60	GO:0007050	Cell cycle arrest	4.29E-07	2.24E-04
GO:0007507	Heart development	9.75E-10	1.77E-06	GO:0006952	Defense response	4.26E-30	4.50E-27	GO:0022402	Cell cycle process	7.63E-07	2.52E-04
GO:0007155	Cell adhesion	3.99E-09	4.85E-06	GO:0006954	Inflammatory response	1.16E-26	8.20E-24	GO:0008219	Cell death	5.86E-06	9.93E-04
GO:0022610	Biological adhesion	3.99E-09	4.85E-06	GO:0009611	Response to wounding	4.72E-26	2.49E-23	GO:0016265	Death	6.77E-06	1.14E-03
GO:0006355	Regulation of transcription, DNA-dependent	1.48E-08	1.35E-05	GO:0002684	Positive regulation of immune system process	6.73E-21	2.84E-18	GO:0006915	Apoptosis	2.68E-05	2.49E-03
GO:0008016	Regulation of heart contraction	2.56E-08	1.87E-05	GO:0001775	Cell activation	7.44E-18	2.62E-15	GO:0007049	Cell cycle	2.79E-05	2.72E-03
GO:0007156	Homophilic cell adhesion	3.02E-08	1.83E-05	GO:0050778	Positive regulation of immune response	1.01E-17	3.05E-15	GO:0012501	Programmed cell death	2.98E-05	3.13E-03
GO:0051252	Regulation of RNA metabolic process	3.65E-08	1.90E-05	GO:0002252	Immune effector process	1.52E-17	4.01E-15				
GO:0048562	Embryonic organ morphogenesis	9.21E-08	4.19E-05	GO:0045321	Leukocyte activation	2.21E-16	5.22E-14				
GO:0007389	Pattern specification process	2.20E-07	8.89E-05	GO:0048584	Positive regulation of response to stimulus	2.88E-15	6.10E-13				
Neighboring area											
Pattern 1				Pattern 2				Pattern 3			
ID	Name	*p*-value	FDR	ID	Name	*p*-value	FDR	ID	Name	*p*-value	FDR
GO:0006355	Regulation of transcription, DNA-dependent	4.16E-08	1.56E-04	GO:0006955	Immune response	2.00E-33	2.16E-30	NA	NA	NA	NA
GO:0051252	Regulation of RNA metabolic process	1.03E-07	1.94E-04	GO:0006954	Inflammatory response	2.39E-14	1.29E-11				
				GO:0006952	Defense response	1.23E-13	4.42E-11				
				GO:0009611	Response to wounding	1.63E-11	4.40E-09				
				GO:0042330	Taxis	2.75E-08	5.94E-06				
				GO:0006935	Chemotaxis	2.75E-08	5.94E-06				
				GO:0009615	Response to virus	9.79E-06	1.75E-03				
				GO:0002526	Acute inflammatory response	2.03E-05	3.12E-03				
				GO:0007626	Locomotory behavior	4.72E-05	6.34E-03				
				GO:0002684	Positive regulation of immune system process	6.48E-05	7.74E-03				

^a^FDR corrections were calculated using the Benjamini-Hochberg procedure

*p-values were calculated using Fischer’s test

On the other hand, in the non-irradiated neighboring lung tissue, we obtained slightly different results (Table 1). The biggest difference was that the number of significantly enriched GO terms (FDR <0.01) was greatly reduced compared with those in the focally irradiated lung regions. For example, only two transcription regulation-related GO terms (GO:0006355 and GO:0051252) were enriched in Pattern 1 in the non-irradiated neighboring lung tissues, whereas 29 GO terms were enriched in the focally irradiated lung tissues. Although Pattern 2 from the non-irradiated regions of lung was still associated with immune responses similar to those of the irradiated lung regions, the number of enriched terms was reduced to 9 from 155 (FDR <0.01). Moreover, we did not observe any significant GO term in Pattern 3 from the non-irradiated neighboring lung tissues (Table 1).

**Fig. 4:**
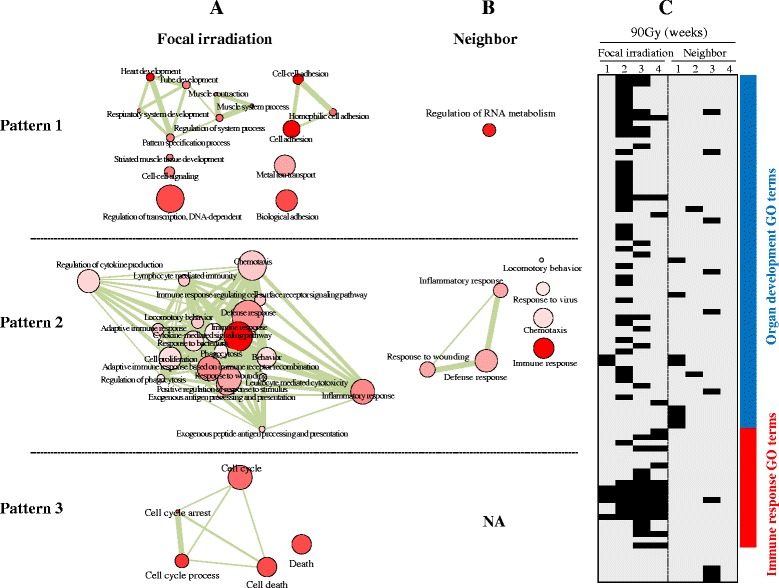
Altered GO terms by focal exposure to high-dose radiation. The network structure among non-redundant GO terms was constructed from all enriched GO terms (FDR <0.01) using the REIVGO program in (**a**) focally-irradiated regions and (**b**) non-irradiated neighboring lung regions. The node size and color intensity are proportional to the hierarchical status and statistical significance of each node, respectively. The edge thickness between nodes represents the closeness of the two nodes. **c** Significantly enriched non-redundant GO terms at each time point were temporally distributed. The columns represent individual samples, while the rows represent statistically significant GO terms (FDR <0.01). The positions of the organ development-related terms and immune-related terms are indicated as bars. A full list of GO terms is depicted in Additional file 6

The GO categories were composed of redundant terms, which had to be eliminated in order to obtain non-redundant GO terms. Figure 4 shows the functional relationship of non-redundant GO terms in the network structure obtained by implementing the REVIGO program. Organ development and morphogenesis-related terms, such as heart development, tube development, and pattern specification processes, were inter-connected with each other in Pattern 1, immune-associated terms were inter-connected in Pattern 2, and cell cycle-related terms were inter-connected in Pattern 3 in tissues from focally-irradiated lung (Fig. 4a), whereas a significantly reduced network structure was observed in the non-irradiated lung tissues (Fig. 4b). This functional segregation according to expressional patterns was also confirmed in a text-based GO terms distribution tree map (Additional file 5). The temporal similarity and differences of biological functions in terms of GO categories between focally-irradiated and non-irradiated neighboring lung regions are compared in Fig. 4c. While organ development-associated terms were enriched early after irradiation, immune response-associated terms were enriched in a temporally delayed fashion, especially in the focally irradiated lung. For a full list of GO terms, see Additional file 6.

As expected from the presence of common genes induced by different doses of radiation, as shown in Additional file 3, 20 Gy radiation also enriched biological functions strongly associated with tissue development from the Down-pattern and the immune response from the Up-pattern (FDR <0.01) (Additional file 7), implying the presence of commonly involved biological functions responding to different doses of radiation. Temporal changes of enriched GO terms also suggest that these two biological changes were effects of both exposures to 20 and 90 Gy high-dose radiation (Additional file 6).

### Pathway analysis

In addition to GO analysis, we examined functional involvement of genes in the temporal patterns using pathway enrichment analysis. Table 2 lists the enriched pathways (FDR <0.01) in Pattern 1, Pattern 2, and Pattern 3 after focal high-dose irradiation. Cardiomyopathy-related pathways (KEGG5414, KEGG5410, and KEGG5412), calcium signaling pathways (KEGG4020), and tight junction pathways (KEGG4530) were significantly enriched in Pattern 1 (FDR <0.01). Immune system-related pathways, such as the cytokine pathway (KEGG4060), systemic lupus erythematosus pathway (KEGG5322), and antigen processing and presentation (KEGG4612), were significantly enriched in Pattern 2 (FDR <0.01). The P53 signaling pathway was the only significant pathway associated with Pattern 3 in the focally irradiated lung tissue (FDR <0.01).

**Table 2:** Pathways enriched in lung focally exposed to high-dose radiation of 90 Gy

Focally irradiated area											
Pattern 1				Pattern 2				Pattern 3			
KEGG ID	Name	*p*-value*	FDR^a^	ID	Name	*p*-value	FDR	ID	Name	*p*-value	FDR
mmu05414	Dilated cardiomyopathy	3.31E-09	6.08E-07	mmu04060	Cytokine-cytokine receptor interaction	5.25E-17	6.78E-15	mmu04115	p53 signaling pathway	4.94E-09	1.78E-07
mmu05410	Hypertrophic cardiomyopathy	1.75E-07	1.61E-05	mmu05322	Systemic lupus erythematosus	2.32E-10	1.50E-08				
mmu05412	Arrhythmogenic right ventricular cardiomyopathy	5.71E-06	3.50E-04	mmu05332	Graft-versus-host disease	1.34E-09	5.78E-08				
mmu04020	Calcium signaling pathway	1.24E-05	5.71E-04	mmu05330	Allograft rejection	1.81E-09	5.83E-08				
mmu00982	Drug metabolism	1.85E-05	6.81E-04	mmu04142	Lysosome	2.10E-09	5.42E-08				
mmu04530	Tight junction	1.15E-04	3.53E-03	mmu04612	Antigen processing and presentation	4.62E-09	9.93E-08				
mmu04360	Axon guidance	1.58E-04	4.14E-03	mmu04940	Type I diabetes mellitus	7.14E-09	1.32E-07				
				mmu05310	Asthma	2.00E-08	3.23E-07				
				mmu04062	Chemokine signaling pathway	7.04E-08	1.01E-06				
				mmu04514	Cell adhesion molecules (CAMs)	1.51E-07	1.94E-06				
				mmu04650	Natural killer cell mediated cytotoxicity	2.78E-07	3.26E-06				
				mmu04672	Intestinal immune network for IgA production	9.21E-07	9.90E-06				
				mmu05340	Primary immunodeficiency	1.10E-05	1.09E-04				
				mmu05320	Autoimmune thyroid disease	1.11E-05	1.03E-04				
				mmu05416	Viral myocarditis	1.25E-05	1.08E-04				
				mmu04660	T cell receptor signaling pathway	1.95E-05	1.57E-04				
				mmu04640	Hematopoietic cell lineage	1.55E-04	1.17E-03				
				mmu04666	Fc gamma R-mediated phagocytosis	5.51E-04	3.94E-03				
				mmu04610	Complement and coagulation cascades	8.61E-04	5.82E-03				
				mmu04621	NOD-like receptor signaling pathway	9.22E-04	5.93E-03				
				mmu04670	Leukocyte transendothelial migration	1.19E-03	7.32E-03				
				mmu04662	B cell receptor signaling pathway	1.43E-03	8.41E-03				
Neighboring area											
Pattern 1				Pattern 2				Pattern 3			
KEGG ID	Name	*p*-value	FDR	ID	Name	*p*-value	FDR	ID	Name	*p*-value	FDR
mmu00982	Drug metabolism	8.90E-06	1.67E-03	mmu04060	Cytokine-cytokine receptor interaction	3.39E-08	2.89E-06	NA	NA	NA	NA
mmu05410	Hypertrophic cardiomyopathy	8.99E-05	8.41E-03	mmu04062	Chemokine signaling pathway	1.36E-05	5.80E-04				
mmu04260	Cardiac muscle contraction	1.36E-04	8.46E-03	mmu04620	Toll-like receptor signaling pathway	2.68E-05	7.58E-04				
mmu04010	MAPK signaling pathway	1.80E-04	8.44E-03								
mmu05414	Dilated cardiomyopathy	1.93E-04	7.24E-03								

^a^FDR corrections were calculated using the Benjamini-Hochberg procedure

*p-values were calculated using Fischer’s test

On the other hand, in the non-irradiated neighboring lung regions, we also observed that cardio-related pathways, such as cardiomyopathy (KEGG5410 and KEGG5414) and cardiac muscle contraction (KEGG4260), were enriched in Pattern 1 and immune-response pathways, such as cytokine receptor pathways (KEGG4060), chemokine signaling (KEGG4062), and Toll-like receptor pathways (KEGG4620), were enriched in Pattern 2, although the number of significant pathways was greatly reduced. The positions of the temporally regulated genes in the pathways are depicted in Additional file 8. Full list of genes associated with enriched pathways in Table 2 is shown in Additional file 2.

Consistent with GO analysis results, exposure to 20 Gy radiation also induced enrichment of cardiomyopathy-related pathways from the Down-pattern and immune response pathway from the Up-pattern, as shown in Additional file 9 (FDR <0.01).

**Fig. 5:**
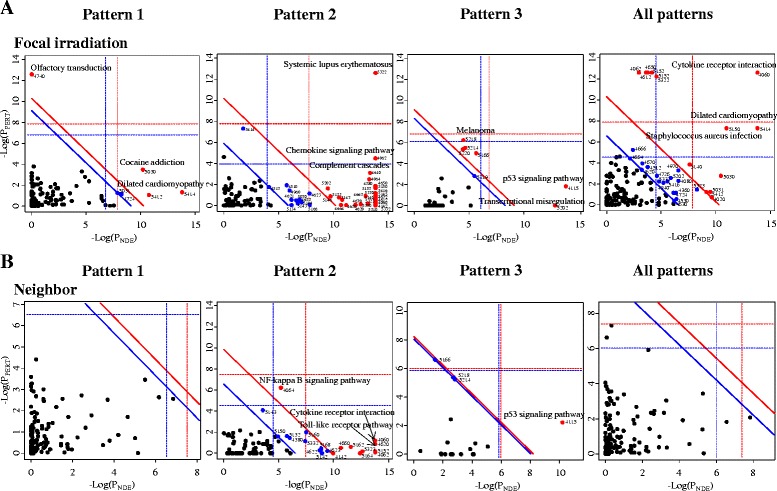
Pathways altered by focal exposure to high-dose radiation. Pathways involved in Pattern 1, Pattern 2, Pattern 3, and all Patterns were analyzed via the Signaling Pathway Impact Analysis (SPIA) program in (**a**) focally-irradiated regions and (**b**) non-irradiated neighboring lung regions. The horizontal axis represents pathway over‐representation (P_NDE_), while the vertical axis indicates pathway perturbation (P_PERT_). The dotted horizontal and vertical lines represent the corrected thresholds (1 %) of significance (*red color* for Bonferroni and blue for FDR correction) for each axis value. The *red* and *blue circles* at the right of the oblique lines are significant pathways with KEGG ID after the correction (*red line* for 1 % and *blue line* for 5 % FDR correction) of the global *p*-values, P_G_. P_G_, representing pathway rank, was calculated from the combined probability of both P_NDE_ and P_PERT_. The list of pathways for the red circles is shown in Table 3

For a more systematic analysis of the pathways, we conducted SPIA pathway analysis, which calculates a significant *p*-value for a pathway using its topology and expressional level. Figure 5 and Table 3 show that four pathways containing dilated cardiomyopathy (KEGG5414) and arrhythmogenic right ventricular cardiomyopathy (KEGG5412) were significant (P_G_ <0.01, FDR <0.01) in Pattern 1 (Fig. 5a), and 29 pathways were mainly implicated with immune-response in Pattern 2 in focally-irradiated areas of lung tissue, consistent with the result of the pathway enrichment analysis (Table 2). In Pattern 3, we observed six pathways that were significant, including the p53 pathway (KEGG4115) and cancer-related pathways (KEGG5202, KEGG5218, KEGG5214, and KEGG5220). However, in the non-irradiated neighboring lung tissue, we did not identify any significant pathways in Pattern 1 or Pattern 3 (Fig. 5b). Only immune response related-pathways were statistically significant in Pattern 2. When all genes included in the three temporal patterns were analyzed together (All patterns in Fig. 5 and Table 3), cardiomyopathy pathways and immune pathways were still significantly enriched only in the focally irradiated regions but not in the neighboring regions. Full list of genes associated with enriched pathways in SPIA is shown in Additional file 2.

**Table 3:** Pathways significantly enriched (FDR <0.01) in lung focally exposed to high-dose radiation of 90 Gy using SPIA pathway analysis

Focally irradiated area															
Pattern 1				Pattern 2				Pattern 3				All patterns			
KEGG ID	Name	P_G_*	FDR^a^	ID	Name	P_G_	FDR	ID	Name	P_G_	FDR	ID	Name	P_G_	FDR
5414	Dilated cardiomyopathy	3.23E-07	4.20E-05	4060	Cytokine-cytokine receptor interaction	1.91E-23	2.21E-21	4115	p53 signaling pathway	7.45E-10	3.43E-08	4060	Cytokine-cytokine receptor interaction	1.81E-12	2.37E-10
5030	Cocaine addiction	1.68E-05	1.09E-03	5150	Staphylococcus aureus infection	1.91E-17	1.11E-15	5202	Transcriptional misregulation in cancer	3.70E-05	8.51E-04	5414	Dilated cardiomyopathy	4.36E-10	2.86E-08
4740	Olfactory transduction	4.54E-05	1.96E-03	5140	Leishmaniasis	5.15E-17	1.99E-15	5218	Melanoma	2.65E-04	3.21E-03	5150	Staphylococcus aureus infection	2.19E-07	9.56E-06
5412	Arrhythmogenic right ventricular cardiomyopathy	9.17E-05	2.98E-03	5322	Systemic lupus erythematosus	8.35E-16	2.42E-14	5166	HTLV-I infection	2.79E-04	3.21E-03	5322	Systemic lupus erythematosus	6.35E-07	2.08E-05
				4380	Osteoclast differentiation	6.38E-15	1.48E-13	5214	Glioma	4.27E-04	3.92E-03	5152	Tuberculosis	9.41E-07	2.47E-05
				4062	Chemokine signaling pathway	7.48E-14	1.45E-12	5220	Chronic myeloid leukemia	6.01E-04	4.61E-03	4650	Natural killer cell mediated cytotoxicity	1.33E-06	2.87E-05
				5152	Tuberculosis	9.50E-14	1.57E-12					4612	Antigen processing and presentation	1.53E-06	2.87E-05
				4142	Lysosome	5.05E-13	7.33E-12					4062	Chemokine signaling pathway	2.85E-06	4.67E-05
				5323	Rheumatoid arthritis	1.07E-12	1.37E-11					5030	Cocaine addiction	2.49E-05	3.62E-04
				4612	Antigen processing and presentation	1.45E-12	1.69E-11					5140	Leishmaniasis	1.33E-04	1.73E-03
				4650	Natural killer cell mediated cytotoxicity	1.94E-10	2.04E-09					5031	Amphetamine addiction	2.48E-04	2.95E-03
				5310	Asthma	1.99E-09	1.92E-08					4020	Calcium signaling pathway	3.57E-04	3.60E-03
				5330	Allograft rejection	2.31E-09	2.07E-08					5412	Arrhythmogenic right ventricular cardiomyopathy	3.57E-04	3.60E-03
				5332	Graft-versus-host disease	2.58E-09	2.14E-08					4970	Salivary secretion	5.69E-04	5.32E-03
				4940	Type I diabetes mellitus	9.98E-09	7.72E-08					5323	Rheumatoid arthritis	6.43E-04	5.61E-03
				4672	Intestinal immune network for IgA production	2.96E-08	2.15E-07								
				5162	Measles	6.19E-08	4.23E-07								
				5164	Influenza A	1.14E-07	7.35E-07								
				5168	Herpes simplex infection	1.68E-07	1.03E-06								
				4660	T cell receptor signaling pathway	5.60E-07	3.25E-06								
				4610	Complement and coagulation cascades	1.29E-06	7.11E-06								
				5145	Toxoplasmosis	2.31E-06	1.22E-05								
				4064	NF-kappa B signaling pathway	2.43E-06	1.22E-05								
				5416	Viral myocarditis	4.68E-06	2.26E-05								
				4662	B cell receptor signaling pathway	1.46E-05	6.77E-05								
				5320	Autoimmune thyroid disease	2.37E-05	1.04E-04								
				4666	Fc gamma R-mediated phagocytosis	2.41E-05	1.04E-04								
				4620	Toll-like receptor signaling pathway	4.70E-05	1.95E-04								
				4670	Leukocyte transendothelial migration	7.56E-05	3.02E-04								
				5146	Amoebiasis	1.45E-04	5.62E-04								
				5142	Chagas disease	1.58E-04	5.90E-04								
				4630	Jak-STAT signaling pathway	1.71E-04	6.21E-04								
				5202	Transcriptional misregulation in cancer	1.81E-04	6.36E-04								
				4664	Fc epsilon RI signaling pathway	2.64E-04	9.01E-04								
				5133	Pertussis	2.87E-04	9.50E-04								
				5414	Dilated cardiomyopathy	1.13E-03	3.65E-03								
				4623	Cytosolic DNA-sensing pathway	1.28E-03	4.03E-03								
Neighboring area															
Pattern 1				Pattern 2				Pattern 3				All patterns			
ID	Name	P_G_	FDR	ID	Name	P_G_	FDR	ID	Name	P_G_	FDR	ID	Name	P_G_	FDR
NA	NA	NA	NA	4060	Cytokine-cytokine receptor interaction	5.49E-10	4.72E-08	4115	p53 signaling pathway	5.40E-05	1.08E-03	NA	NA	NA	NA
				4620	Toll-like receptor signaling pathway	4.06E-08	1.74E-06								
				4062	Chemokine signaling pathway	2.81E-07	8.06E-06								
				5152	Tuberculosis	1.75E-06	3.77E-05								
				5323	Rheumatoid arthritis	3.60E-05	6.20E-04								
				5164	Influenza A	5.34E-05	7.65E-04								
				5162	Measles	6.80E-05	8.36E-04								
				4064	NF-kappa B signaling pathway	1.35E-04	1.45E-03								
				4660	T cell receptor signaling pathway	1.61E-04	1.54E-03								
				4142	Lysosome	5.08E-04	4.37E-03								
				4623	Cytosolic DNA-sensing pathway	6.69E-04	5.23E-03								
				5160	Hepatitis C	7.90E-04	5.65E-03								
				5168	Herpes simplex infection	9.39E-04	6.21E-03								
				4622	RIG-I-like receptor signaling pathway	1.21E-03	7.17E-03								
				5142	Chagas disease	1.25E-03	7.17E-03								
				5332	Graft-versus-host disease	1.79E-03	9.66E-03								

^a^FDR value is calculated for P_G_ value

*P_G_ value indicates the global pathway significance *p* value, which combines the enrichment *p* values and the perturbation *p* values in regard to pathway topology with a random bootstrap iteration number of 3000

**Fig. 6:**
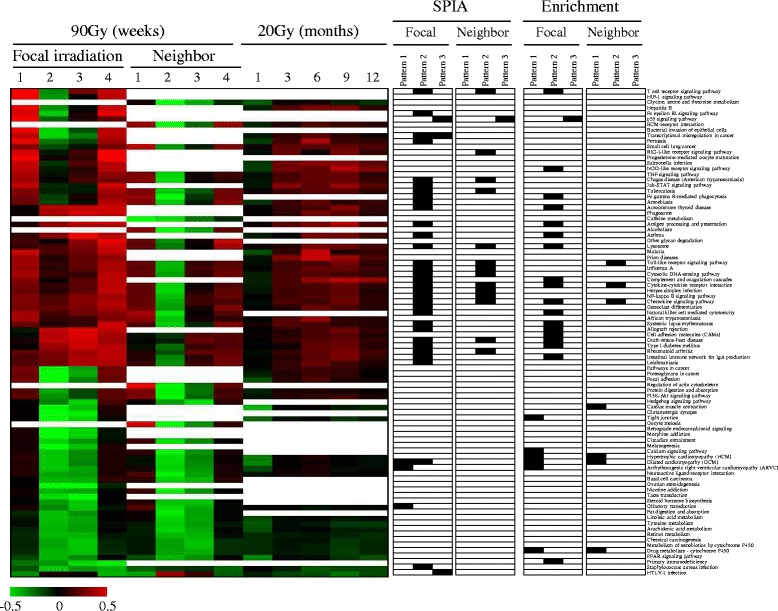
Pathway activities temporally changed by irradiation. Pathway activities were calculated by linearly combining gene expression levels and then were hierarchically clustered. The columns represent individual samples and the rows represent the pathways. The red and green colors reflect high and low activity levels, respectively, as indicated by the scale bar with arbitrary units. Pathways enriched from SPIA and simple enrichment analysis (FDR <0.01) are highlighted in black at the right panel with names

In addition to the identification of enriched pathways, we also measured temporal changes of the pathways’ activities by linearly combining the expression values of all genes into an activity index. Figure 6 shows the temporal changes of 88 pathways (FDR <0.01) in the focally irradiated regions and their neighboring regions. The positions of the pathways selected from SPIA analysis (Table 3) and simple pathway enrichment analysis (Table 2) were also compared in parallel. Consistent with the previous results, cardiomyopathy-related pathways and the calcium-signaling pathway from Pattern 1 were down-regulated in both the focally irradiated regions and non-irradiated neighboring lung regions. In addition to these pathways, many other pathways were also down-regulated, especially at two and three weeks in both lung regions after exposure to high-dose radiation. On the other hand, immune-related pathways, especially those enriched in Pattern 2, were more activated in focally irradiated regions than non-irradiated neighboring lung regions. Moreover, although Pattern 2 was generally composed of genes showing temporally increased expression, pathway activities enriched in Pattern 2 in the neighboring lung regions were down-regulated, especially two weeks after irradiation. These results might indicate the importance of other genes that were not included in the temporal pattern for the determination of the overall activity of the pathway. Interestingly, exposure to 20 Gy radiation displayed a similar temporal pattern of pathway activities to focal exposure to 90 Gy (Fig. 6). In particular, many immune-related pathways were also up-regulated after 20 Gy irradiation.

### Module-based network analysis

Co-expression of functionally associated genes suggested the presence of a co-related network of genes induced by radiation. Although pathway information provided one of these kinds of networks, we expanded it to the more comprehensive functional interaction network constructed by Wu et al., which includes protein-protein interactions, gene co-expression, protein domain interaction, GO annotations, and text-mined protein interactions [23].

**Fig. 7:**
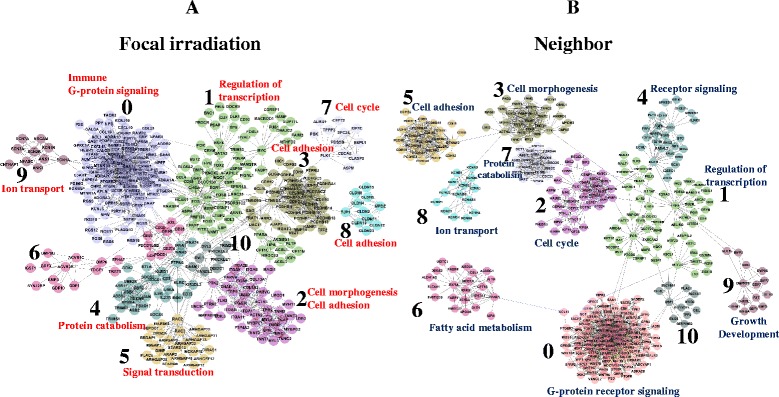
Interaction network of genes induced by focal exposure to high-dose radiation. By implementing Reactome FI application, an interaction network was constructed among 6681 and 6799 differentially expressed genes with at least two-fold variation in (**a**) focally-irradiated regions and in (**b**) non-irradiated neighboring lung regions, respectively. Both networks from (**a**) and (**b**) are composed of a total of 11 modules (as indicated from 0 to 10), each of which is differently colored. The representative GO term associated with each module was obtained from REVIGO program using module genes (FDR <0.01)

Using genes with at least two-fold differential expression (6681 genes from focally-irradiated regions and 6769 genes from non-irradiated neighboring lung regions), we constructed a network of genes with Pearson correlation coefficients greater than 0.8, as shown in Fig. 7. Networks consisted of 11 inter-connected modules (from 0 to 10) in both focally irradiated (Fig. 7a) and non-irradiated neighboring lung regions (Fig. 7b). Representative biological functions associated with each module (FDR <0.01) are also depicted. As observed in the previous GO and pathway analyses, organ development-related functions, such as morphogenesis and cell adhesion, were enriched in modules 2, 3, and 8, and immune response function was enriched in module 0 in the focally irradiated lung regions. In addition, cell cycle function was enriched in module 7. Similar functions were also associated with each module from non-irradiated neighboring lung regions. For example, cell morphogenesis and cell cycle functions were associated with modules 3 and 2, respectively (Fig. 7b). A full list of GO terms associated with each module and their functional network structure are shown in Additional file 10. Individual genes included in each module from the whole network are listed in Additional file 11 with detailed network characteristics.

The similarity of networks between the two regions of lung was also confirmed by comparison of module composition. As shown in Additional file 12, many genes included in each module from focally irradiated lung, especially major modules, such as modules 0, 2, and 3, were also distributed in modules from non-irradiated lung tissues. However, some minor modules, such as modules 5, 8, 9, and 10, were unique.

This result supports the similarity and distinction occurring in the two different areas of lung and suggests biological functions regulated by radiation, such as organ development, immune response, and cell cycle, are closely interrelated.

## Discussion

Side effects occur in 5 to 15 % of people who receive radiation therapy for lung cancer. Although SBRT is well tolerated in medically inoperable NSCLC patients, radiation pneumonitis remains a problem for many lung cancer patients [1]. Because pneumonitis is significantly associated with the conformity index, it is important that the radiation beam be tightly focused on the target region of tumor in order to avoid unnecessary exposure of normal lung tissue to high-dose radiation. Previously, we developed an animal model system that can deliver focal high-dose radiation to mouse lung with a minimum diameter of 1 mm [12]. Our results using this SBRT mouse model demonstrated that non-irradiated lung regions had a similar gene expression pattern to the pattern observed in the focally irradiated lung regions. Given that the distance of the non-irradiated neighboring region from the focally irradiated region is critical to the homogeneity of the neighboring tissue, we attempted to obtain neighboring regions as far as possible from the focally irradiated region. However, visual confirmation and changes in gene expression in this study provide evidence of the development of fibrosis even in non-irradiated neighboring lung regions. There are two possibilities for this phenomenon: a direct and an indirect mechanism. The direct explanation is that the diffuse radiation beam could have irradiated the boundary regions of the focally irradiated spot and affected gene expression directly in the non-irradiated area. The indirect explanation is that substances secreted from the focally irradiated regions might have induced pathological and expressional changes in the non-irradiated regions of lung. This hypothesis was supported by the observation that fibrotic changes of the boundary regions were delayed, occurring three weeks after irradiation, whereas fibrotic changes occurred two weeks after irradiation in the focally irradiated lung areas. We believe these direct and indirect effects of radiation may combine to induce cellular and pathological responses in non-irradiated lung regions.

Closer inspection of the gene expression patterns demonstrated that three sub-classes of temporal patterns were induced by focal exposure to high-dose radiation in both directly irradiated and non-irradiated neighboring lung regions. The first subclass was composed of a set of down-regulated genes (Pattern 1) and was strongly associated with organ development-related functions, including cell adhesion, organ development, and organ morphogenesis (FDR <0.01). The second subclass, which was composed of an up-regulated gene set (Pattern 2), included immune-response functions, such as defense response, inflammatory response, and wound response (FDR <0.01). The third subclass, which was composed of a set of initially up-regulated genes (Pattern 3), was associated with cell cycle-related functions, such as the p53 signaling pathway, cell cycle arrest, and apoptosis (FDR <0.01). Despite the overall similarity of gene expression and their associated biological functions, the directly exposed regions were clearly more associated with the above-mentioned biological functions than the non-irradiated neighboring lung regions. Again, this result demonstrates that even when radiation is precisely concentrated on a focal region, there is the potential for neighboring lung regions to be affected.

A previous report demonstrated that radiation can disrupt the integrity of lung epithelial and endothelial tissues, which results in edema, recruitment of leukocytes, angiogenesis, and diverse biological events [24]. In addition, radiation can modulate focal adhesion-associated proteins [25] and induce gross structural changes in the lung leading to proteinaceous fluid leaking into alveolar spaces [26, 27]. These previous reports provide evidence that irradiation can de-regulate the integrity of lung, as seen in our study in the Pattern 1 genes, which were involved in cell adhesion and organ morphogenesis. Changes of cell adhesion functions are widely known in the process of tumor progression and metastasis in lung cancer in which tumor microenvironment such as stroma acts as the key components in the regulation of mobility of lung cancer cells [28, 29]. Therefore our results strongly supports the importance of lung stromal changes in the process of radiation responses as well as tumorigenesis. Moreover, morphological changes in irradiated lung tissues including stroma might be one of the markers of the presence of the epithelial/endothelial-mesenchymal transition (EMT/EndMT) process, which has been demonstrated in human idiopathic and experimental pulmonary fibrosis [30–32]. Although ionizing radiation was reported to induce EMT-like changes in lung epithelial cell lines, its role in radiation-induced lung injury has not been established in vivo.

On the other hand, it has been reported that proteins leaking from the vascular system into the extravascular space is a hallmark of pulmonary inflammation and contributes to acute lung injury [33] during which edemagenic agonists, such as thrombin and histamine, disrupt junctional interactions among endothelial cells [34]. In addition, thorax irradiation has been reported to trigger the recruitment of various immune cells into the lung, including monocytes, neutrophils, basophils and lymphocytes, that are responsible for local and systemic expression of cytokines and chemokines after radiation exposure [4, 35]. Consistent with these previous reports, we also measured that the temporally increased Pattern 2 genes were predominantly associated with the immune response. Taken together, de-regulation of pulmonary integrity by radiation could result in increased vascular permeability followed by recruitment of inflammatory mediators into the irradiated region of lung. We also hypothesize that leaked molecules from directly-irradiated lung regions might induce similar, but attenuated, responses in non-irradiated neighboring lung regions. In addition, we tried to identify differentially expressed genes between directly-irradiated lung regions and non-irradiated neighboring lung regions. However, only a handful of genes (4 genes with FDR <0.01) were differentially expressed (Additional file 13). With these genes, we could not obtain statistically significant biological functions differentially associated between two lung regions. In other words, this results represents the similarity of two types of lung regions in response to direct or indirect irradiation.

Intriguingly, cardiomyopathy-related pathways were prominently enriched by focal irradiation in our study. Recently, it was also reported that organ morphogenesis and muscle contraction functions are significantly associated with cardiomyopathy progression in gene expression profile and network module analyses [36]. Moreover, heart failure, such as cardiomyopathy, is known to induce cardiac tissue remodeling processes involving changes of tissue structure and cardiac function [37, 38]. Considering these previous reports, enrichment of cardiomyopathy-related pathways in our study was thought to result from the tissue morphogenic activity induced by high-dose irradiation.

In addition to the two major patterns of gene expression (Pattern 1 and Pattern 2), we were able to characterize a small group genes that were initially up-regulated 1 week after irradiation (Pattern 3), and this pattern was significantly associated with p53 signal transduction, cell cycle arrest, and apoptosis. Because DNA is a critical cellular target of radiation, various forms of DNA damage can be induced by radiation exposure. The immediate response to damaged DNA is the stimulation of DNA repair machinery and the activation of cell cycle checkpoints, followed by down-stream cellular responses, such as apoptosis [39]. Therefore, our results implicate possible DNA damage and activation of repair systems, such as the p53 signaling pathway, in the lung early after radiation exposure. This initial response of pathways by focal irradiation could be related with acute lung injury by inducing immune response of Pattern 2. Actually, p53 response by radiation was reported to be related with immune reaction in lymphoma [40, 41].

In addition to biological functions mentioned in detail here, many diverse pathways or GO terms also responded to radiation exposure, as shown in Figs. 4 and 6. Interestingly, these diverse biological processes induced by radiation were inter-connected with each other as shown in the functional network structure in which organ development-related functions, immune response, cell cycle, and signal transduction functions were connected via modules (Fig. 7). Because modules in networks were composed of co-interacting proteins, the inter-connection of modules represents the presence of concerted regulatory mechanisms involving sequential changes in biological activities. For example, G-proteins such as GNB5 and GNG3, which were core nodes with ~90° in module 0 of focally-irradiated lung areas, have proven to be associated with organ development of brain or central nervous system [42–44]. JUN and FOS, core nodes in module 1 of focally-irradiated lung areas, are transcription factors involved in diverse cellular processes such as development and immune process [45, 46]. CTNNB1, core node with degrees of 51 in module 3 of focally-irradiated lung areas, is a key transcriptional regulator and is involved in morphogenesis or developmental process [47, 48]. CTNNB1 was reported to regulate immune response [49] and to integrate cell adhesion and differentiation [50]. Also PTPN6, module 4 core node, can affect the B cell development [51] and T-cell signaling [52]. These reports supports the inter-connection of diverse biological functions induced by irradiation.

Finally, we attempted to simulate conventional radiotherapy in our mouse model by exposing a wide lung region (7 mm in diameter of whole left lung) to 20 Gy radiation. As expected, low-dose radiation did not induce prominent changes in gene expression in the gene expression profile overall compared to the genes induced by high-dose (90 Gy) radiation. However, two small groups of genes that initially showed a down-regulatory pattern (Down-pattern) and temporally up-regulatory pattern (Up-pattern) partially overlapped with Patterns 1 and 2, respectively. Moreover, biological functions associated with these two patterns were similar to those associated with high-dose radiation. Organ development-related and immune response functions were significantly associated with the Down-pattern and Up-pattern, respectively. In particular, delayed activation of immune response functions was consistent with a previous report that demonstrated pulmonary fibrosis develops between 6 and 24 months post-irradiation [4]. Our results indicate that despite the overall differences in gene expression, there might be common biological functions that occur in response to different doses of radiation.

Previous reports have demonstrated that SBRT using hypo-fractionated radiotherapy can overcome the radiation-induced lung injury that is commonly induced by conventional radiotherapy [1, 2]. However, we were not able to conclude whether focal, high-dose irradiation can reduce side-effects of radiation that occur in normal lung regions, because expression of significant numbers of genes was also changed in the non-irradiated lung regions. Additionally, fibrotic change was evident in non-irradiated regions in a temporally delayed fashion. Therefore, the molecular pathological changes occurring in the non-irradiated neighboring region after focal exposure to high-dose radiation require further study. Furthermore, the combined effect of radiation dose and elapsed time after irradiation on gene expression also needs to be clarified.

## Conclusion

In this study, we observed focal exposure to high-dose radiation to cause temporal changes in the expression of genes associated with organ development and the immune response in both directly irradiated regions and non-irradiated neighboring lung regions in an SBRT mouse model. Our results contribute to an understanding of tissue toxicity, including radiation pneumonitis and fibrosis, that occurs in small-cell lung cancer patients treated with radiotherapy.

## Availability of supporting data

The data sets supporting the results of this article are included within the article and its additional files. Microarray data are available in the Gene Expression Omnibus (http://www.ncbi.nlm.nih.gov/geo) under accession number GSE60541.

## Additional files

Additional file 1:
**Experimental confirmation of expression of genes included in each pattern after focal exposure to high-dose radiation of 90 Gy in the lung tissue.** (A) Expression of genes included in each pattern were confirmed in triplicate by quantitative real-time PCR. Each mRNA expression was normalized to GAPDH and presented in mean ± standard deviation, in which statistical significance was measured using t-test. (B) Primers used in RT-PCR (C) Immunohistochemical staining of CDKN1A was performed in focally irradiated lungs. Positively stained cells were counted in representative images of immunohistochemistry (×200). Statistical significance was measured using t-test by comparing counts from irradiated samples with those from non-irradiated control. (PDF 607 kb) Additional file 2:
**The full list of genes include in each pattern and associated with enriched pathways.** (XLSX 1669 kb) Additional file 3:
**Temporal gene expression patterns after exposure to 20 Gy radiation.** Temporally altered genes were identified by the Short Time-series Expression Miner (STEM) analysis in mouse lung. Genes were classified into two patterns (FDR <0.001). (A) Down-pattern and (B) Up-pattern comprised 147 and 145 genes, respectively. These genes were compared with genes obtained from focally exposed (90Gy) regions and non-irradiated neighboring lung regions in a Venn diagram. (PDF 138 kb) Additional file 4:
**GO enrichment analysis in lung focally exposed to high-dosage radiation of 90 Gy.** (PDF 261 kb) Additional file 5:
**Tree distribution of GO terms altered by focal exposure to high-dose radiation.** Tree structures composed of non-redundant GO terms were constructed using the REIVGO program from all enriched GO terms (FDR <0.01) in focally irradiated regions and non-irradiated neighboring lung regions. In each tree structure, closely related terms are presented in the same color. The size of each GO term is proportional to the level of statistical significance. (PDF 238 kb) Additional file 6:
**GO terms altered by focal exposure to 90 Gy and diffused exposure to 20 Gy.** Significantly enriched non-redundant GO terms at each time point were temporally distributed. The columns represent individual samples, while the rows represent statistically significant GO terms (FDR <0.01). The positions of the organ development-related terms and immune-related terms are indicated as bars. (PDF 121 kb) Additional file 7:
**GO enrichment analysis in lung exposed to low-dosage radiation of 20 Gy.** (PDF 195 kb) Additional file 8:
**Top five pathways enriched in Pattern 1, Pattern 2, and Pattern 3 in focally irradiated regions and non-irradiated neighboring lung regions.** The position of each gene in the pathways is colored red for the focally irradiated regions, blue for the non-irradiated neighboring lung regions, and yellow for both regions. The image of each pathway was adopted from KEGG homepage (http://www.kegg.jp). (PDF 656 kb) Additional file 9:
**Pathway enrichment analysis in lung exposed to low-dosage radiation of 20 Gy.** (PDF 192 kb) Additional file 10:
**Network structure of GO terms associated with modules from (A) focally-irradiated (90 Gy) regions and (B) non-irradiated neighboring lung regions.** The network structure among non-redundant GO terms was constructed from all enriched GO terms (FDR <0.01) using the REIVGO program. The node size and color intensity are proportional to the hierarchical status and statistical significance of each node, respectively. The edge thickness between nodes represents the closeness of two nodes. (PDF 211 kb) Additional file 11:
**Genes included in each module of network structure.** (PDF 363 kb) Additional file 12:
**Comparison of module composition between two network structures obtained from focally irradiated regions and non-irradiated neighboring lung regions.** Both network structures were composed of 11 modules. Each spot represents a common gene present between modules. (PDF 272 kb) Additional file 13:
**Differentially expressed genes between focally-irradiated regions and non-irradiated neighboring lung regions.** Genes were selected using two classes times-series analysis implemented in Significance Analysis of Microarray (SAM) program [53]. Dotted lines represents the statistical significance level of FDR <0.01. Red circles represents over-expressed genes in focally-irradiated lung regions. (PDF 152 kb)

## Abbreviations

DAVID: database for annotation, visualization, and integrated discovery
ECM: extracellular matrix
EMT: epithelial mesenchymal transition
FDR: false discovery rate
GO: gene ontology
KEGG: Kyoto Encyclopedia of genes and genomes
NSCLC: non-small-cell lung cancer
SBRT: stereotactic body radiotherapy
SPIA: signaling pathway impact analysis
STEM: short time series expression miner
