# Supplementary material for: Focal exposure of limited lung volumes to high-dose irradiation down-regulated organ development-related functions and up-regulated the immune response in mouse pulmonary tissues
Source: BMC Genet. 2016 Jan 27;17:29. doi: 10.1186/s12863-016-0338-9 (PMC4729165; doi:10.1186/s12863-016-0338-9)
Supplement: Additional file 1: — Experimental confirmation of expression of genes included in each pattern after focal exposure to high-dose radiation of 90 Gy in the lung tissue. (A) Expression of genes included in each pattern were confirmed in triplicate by quantitative real-time PCR. Each mRNA expression was normalized to GAPDH and presented in mean ± standard deviation, in which statistical significance was measured using t-test. (B) Primers used in RT-PCR (C) Immunohistochemical staining of CDKN1A was performed in focally irradiated lungs. Positively stained cells were counted in representative images of immunohistochemistry (×200). Statistical significance was measured using t-test by comparing counts from irradiated samples with those from non-irradiated control. (PDF 607 kb) [file 12863_2016_338_MOESM1_ESM.pdf]

Additional file 2

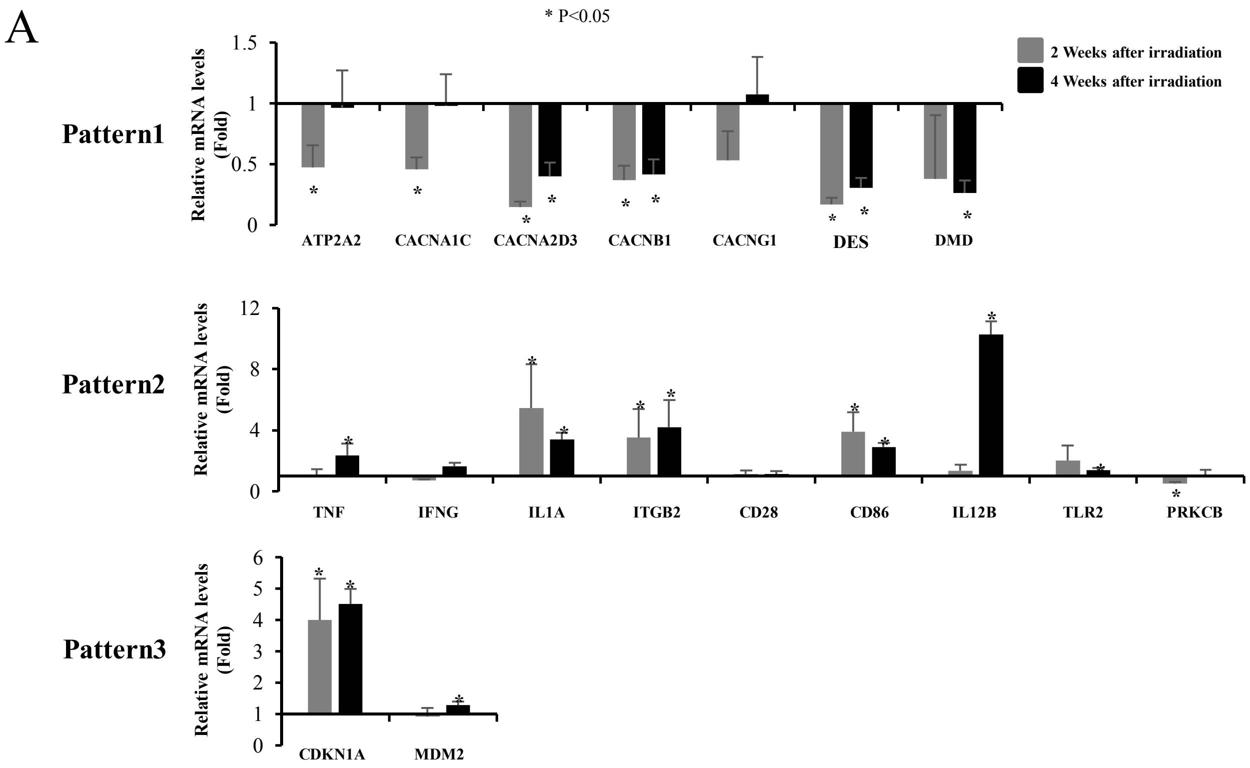

B

	Gene name	Sequence
Pattern1	ATP2A2	Forward: GTCCTGTTCTGCCGCATAGT
		Reverse: GGAGACTTGGCTGATGGCTT
	CACNA1C	Forward: AAGGAGCTGGATTCGACGTG
		Reverse: CATCCATGCCCTCGTCTTCG
	CACNA2D3	Forward: ACTGAACGAGGCCTTCAACA
		Reverse: GCAAGCTTTTGAGCCTGAGGG
	CACNB1	Forward: TGACACCATCAACCACCCAG
		Reverse: GACCAGAGGGACATCGGCTA
	CACNG1	Forward: CTGCGCATTTCTGTCCTTCG
		Reverse: AGGATTCCCAGGGGTTCTGA
	DES	Forward: CCATTGCCCTGGGATGAACT
		Reverse: AGGACTGTCCCCATCCCTAC
	DMD	Forward: TGGCTTTTGTGACCAGCGTA
		Reverse: AGGAGACTGTTTGCATCTGGG
Pattern2	TNF	Forward: TAGCCCACGTCGTAGCAAAC
		Reverse: ACCCTGAGCCATAATCCCCT
	IFNG	Forward: CGGCACAGTCATTGAAAGCC
		Reverse: TGCATCCTTTTTCGCCTTGC
	IL1A	Forward: GCGCTTGAGTCGGCAAAGAA
		Reverse: ACAGACTGTCAGCACTTCCC
	ITGB2	Forward: ATGCACCAAGTACAAAGTCAGCAG
		Reverse: AGTGAAGTTCAGCTTCTGGCA
	CD28	Forward: GCCTTTCGCTCTCGGTATGA
		Reverse: TGGCATGCTCGGTACCAAAT
	CD86	Forward: CTTACGGAAGCACCCACGAT
		Reverse: TGTAAATGGGCACGGCAGAT
	IL12B	Forward: GGGACCAGGCCCTATTATGC
		Reverse: GACCAAAGCCAGCTCCTCAT
	TLR2	Forward: CAGTCTTCCTAGGCTGGTGC
		Reverse: AAGGAAACAGTCCGCACCTC
	PRKCB	Forward: CGAGTGACAGCCCAGCTACG
		Reverse: TCCATACAGCAGCGATCCAC
Pattern3	CDKN1A	Forward: GCAGATCCACAGCGATATCCA
		Reverse: TTTCGGCCCTGAGATGTTCC
	MDM2	Forward: CCAGCTTCGGAACAAGAGACT
		Reverse: GATCACTCCCACCTTCAGGC
	qGAPDH	Forward: ACTGTGGTCATGAGCCCTTC
		Reverse: GGGTGTGAACCACGAGAAAT

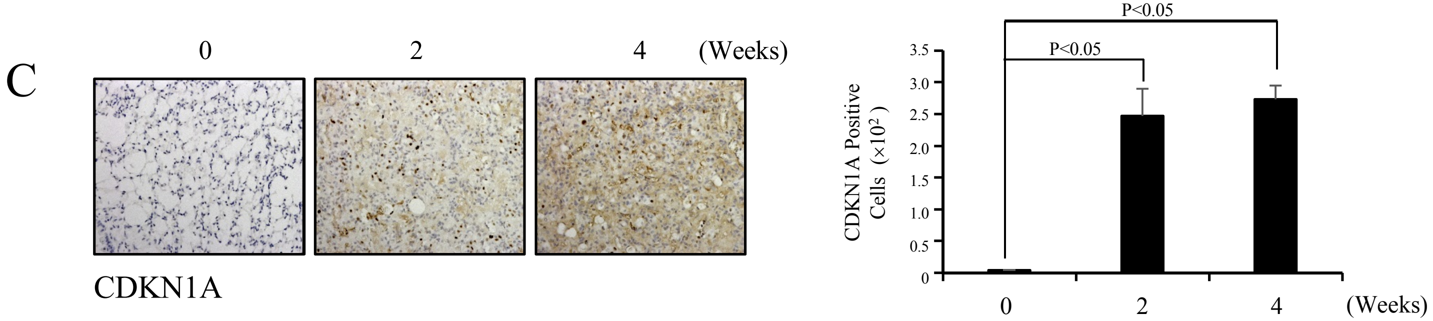

Additional file 2. Experimental confirmation of expression of genes included in each pattern after focal exposure to high-dose radiation of 90 Gy in the lung tissue. (A) Expression of genes included in each pattern were confirmed in triplicate by quantitative real-time PCR. Each mRNA expression was normalized to GAPDH and presented in mean ± standard deviation, in which statistical significance was measured using t-test. (B) Primers used in RT-PCR (C) Immunohistochemical staining of CDKN1A was performed in focally irradiated lungs. Positively stained cells were counted in representative images of immunohistochemistry (×200). Statistical significance was measured using t-test by comparing counts from irradiated samples with those from non-irradiated control.
