# Supplementary material for: Focal exposure of limited lung volumes to high-dose irradiation down-regulated organ development-related functions and up-regulated the immune response in mouse pulmonary tissues
Source: BMC Genet. 2016 Jan 27;17:29. doi: 10.1186/s12863-016-0338-9 (PMC4729165; doi:10.1186/s12863-016-0338-9)
Supplement: Additional file 3: — Temporal gene expression patterns after exposure to 20 Gy radiation. Temporally altered genes were identified by the Short Time-series Expression Miner (STEM) analysis in mouse lung. Genes were classified into two patterns (FDR <0.001). (A) Down-pattern and (B) Up-pattern comprised 147 and 145 genes, respectively. These genes were compared with genes obtained from focally exposed (90Gy) regions and non-irradiated neighboring lung regions in a Venn diagram. (PDF 138 kb) [file 12863_2016_338_MOESM3_ESM.pdf]

Additional file 3

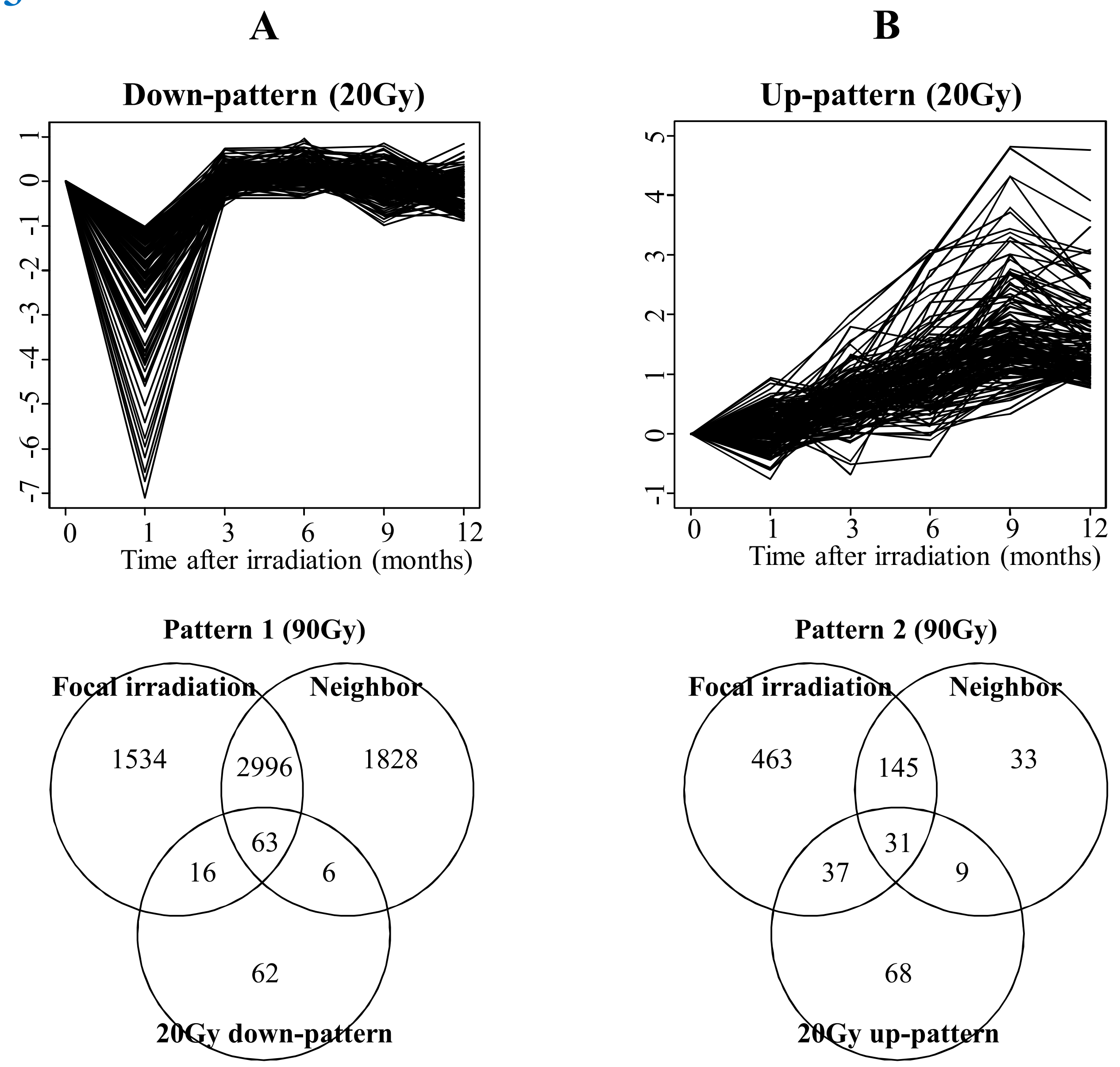

Additional file 3. Temporal gene expression patterns after exposure to 20 Gy radiation. Temporally altered genes were identified by the Short Time-series Expression Miner (STEM) analysis in mouse lung. Genes were classified into two patterns (FDR<0.001). (A) Down-pattern and (B) Up-pattern comprised 147 and 145 genes, respectively. These genes were compared with genes obtained from focally exposed (90Gy) regions and non-irradiated neighboring lung regions in a Venn diagram.
