# Supplementary material for: Focal exposure of limited lung volumes to high-dose irradiation down-regulated organ development-related functions and up-regulated the immune response in mouse pulmonary tissues
Source: BMC Genet. 2016 Jan 27;17:29. doi: 10.1186/s12863-016-0338-9 (PMC4729165; doi:10.1186/s12863-016-0338-9)
Supplement: Additional file 4: — GO enrichment analysis in lung focally exposed to high-dosage radiation of 90 Gy. (PDF 261 kb) [file 12863_2016_338_MOESM4_ESM.pdf]

Additional file 4. GO enrichment analysis in lung focally exposed to high-dosage radiation of 90 Gy

Focally irradiated area

Pattern 1				Pattern 2				Pattern 3			
ID	Name	p-value*	FDR**	ID	Name	p-value	FDR	ID	Name	p-value	FDR
GO:0016337	Cell-cell adhesion	7.33E-10	2.67E-06	GO:0006955	Immune response	2.64E-63	5.58E-60	GO:0007050	Cell cycle arrest	4.29E-07	2.24E-04
GO:0007507	Heart development	9.75E-10	1.77E-06	GO:0006952	Defense response	4.26E-30	4.50E-27	GO:0022402	Cell cycle process	7.63E-07	2.52E-04
GO:0007155	Cell adhesion	3.99E-09	4.85E-06	GO:0006954	Inflammatory response	1.16E-26	8.20E-24	GO:0008219	Cell death	5.86E-06	9.93E-04
GO:0022610	Biological adhesion	3.99E-09	4.85E-06	GO:0009611	Response to wounding	4.72E-26	2.49E-23	GO:0016265	Death	6.77E-06	1.14E-03
GO:0006355	Regulation of transcription, DNA-dependent	1.48E-08	1.35E-05	GO:0002684	Positive regulation of immune system process	6.73E-21	2.84E-18	GO:0006915	Apoptosis	2.68E-05	2.49E-03
GO:0008016	Regulation of heart contraction	2.56E-08	1.87E-05	GO:0001775	Cell activation	7.44E-18	2.62E-15	GO:0007049	Cell cycle	2.79E-05	2.72E-03
GO:0007156	Homophilic cell adhesion	3.02E-08	1.83E-05	GO:0050778	Positive regulation of immune response	1.01E-17	3.05E-15	GO:0012501	Programmed cell death	2.98E-05	3.13E-03
GO:0051252	Regulation of RNA metabolic process	3.65E-08	1.90E-05	GO:0002252	Immune effector process	1.52E-17	4.01E-15				
GO:0048562	Embryonic organ morphogenesis	9.21E-08	4.19E-05	GO:0045321	Leukocyte activation	2.21E-16	5.22E-14				
GO:0007389	Pattern specification process	2.20E-07	8.89E-05	GO:0048584	Positive regulation of response to stimulus	2.88E-15	6.10E-13				
GO:0044057	Regulation of system process	2.41E-06	8.77E-04	GO:0002253	Activation of immune response	1.21E-14	2.33E-12				
GO:0060541	Respiratory system development	4.64E-06	1.53E-03	GO:0002443	Leukocyte mediated immunity	2.08E-14	3.66E-12				
GO:0048568	Embryonic organ development	5.55E-06	1.68E-04	GO:0019882	Antigen processing and presentation	2.62E-14	4.26E-12				
GO:0007423	Sensory organ development	5.79E-06	1.61E-03	GO:0002768	Immune response-regulating cell surface receptor signaling pathway	1.41E-13	2.13E-11				
GO:0048598	Embryonic morphogenesis	6.87E-06	1.78E-03	GO:0002764	Immune response-regulating signal transduction	1.99E-13	2.81E-11				
GO:0007517	Muscle organ development	9.03E-06	2.19E-03	GO:0002694	Regulation of leukocyte activation	2.33E-13	3.08E-11				
GO:0007267	Cell-cell signaling	1.44E-05	3.26E-03	GO:0050865	Regulation of cell activation	3.27E-13	4.06E-11				
GO:0035295	Tube development	1.50E-05	3.19E-03	GO:0042110	T cell activation	1.60E-12	1.88E-10				
GO:0014706	Striated muscle tissue development	2.23E-05	4.49E-03	GO:0051249	Regulation of lymphocyte activation	2.56E-12	2.84E-10				
GO:0030323	Respiratory tube development	2.27E-05	4.33E-03	GO:0045087	Innate immune response	2.70E-12	2.85E-10				
GO:0043583	Ear development	2.76E-05	5.01E-03	GO:0042330	Taxis	4.89E-12	4.92E-10				
GO:0030001	Metal ion transport	3.21E-05	5.54E-03	GO:0006935	Chemotaxis	4.89E-12	4.92E-10				
GO:0048738	Cardiac muscle tissue development	3.48E-05	5.74E-03	GO:0002757	Immune response-activating signal transduction	1.10E-11	1.05E-09				
GO:0030324	Lung development	3.74E-05	5.90E-03	GO:0046649	Lymphocyte activation	1.10E-11	1.01E-09				
GO:0030239	Myofibril assembly	3.88E-05	5.87E-03	GO:0002429	Immune response-activating cell surface receptor	1.29E-11	1.13E-09				

					signaling pathway						
GO:0003012	Muscle system process	4.91E-05	7.12E-03	GO:0048002	Antigen processing and presentation of peptide antigen	2.93E-11	2.48E-09				
GO:0060537	Muscle tissue development	5.35E-05	7.46E-03	GO:0002478	Antigen processing and presentation of exogenous peptide antigen	3.61E-11	2.93E-09				
GO:0042471	Ear morphogenesis	6.23E-05	8.35E-03	GO:0002504	Antigen processing and presentation of peptide or polysaccharide antigen via MHC class II	4.14E-11	3.24E-09				
GO:0006936	Muscle contraction	6.46E-05	8.36E-03	GO:0002460	Adaptive immune response based on somatic recombination of immune receptors built from immunoglobulin superfamily domains	6.47E-11	4.88E-09				
				GO:0002250	Adaptive immune response	6.47E-11	4.88E-09				
				GO:0002449	Lymphocyte mediated immunity	9.75E-11	7.11E-09				
				GO:0002696	Positive regulation of leukocyte activation	1.38E-10	9.76E-09				
				GO:0050863	Regulation of T cell activation	1.40E-10	9.52E-09				
				GO:0050867	Positive regulation of cell activation	2.03E-10	1.34E-08				
				GO:0002495	Antigen processing and presentation of peptide antigen via MHC class II	3.50E-10	2.24E-08				
				GO:0019886	Antigen processing and presentation of exogenous peptide antigen via MHC class II	3.50E-10	2.24E-08				
				GO:0019884	Antigen processing and presentation of exogenous antigen	4.95E-10	3.08E-08				
				GO:0050870	Positive regulation of T cell activation	1.40E-09	8.46E-08				
				GO:0007626	Locomotory behavior	2.10E-09	1.23E-07				
				GO:0001817	Regulation of cytokine production	3.13E-09	1.79E-07				
				GO:0002521	Leukocyte differentiation	3.59E-09	1.99E-07				
				GO:0051251	Positive regulation of lymphocyte activation	3.92E-09	2.13E-07				
				GO:0016064	Immunoglobulin mediated immune response	4.19E-09	2.22E-07				
				GO:0002889	Regulation of immunoglobulin mediated immune response	6.13E-09	3.16E-07				
				GO:0002712	Regulation of B cell mediated immunity	6.13E-09	3.16E-07				
				GO:0019724	B cell mediated immunity	6.69E-09	3.37E-07				
				GO:0002819	Regulation of adaptive immune response	7.61E-09	3.74E-07				
				GO:0002822	Regulation of adaptive immune response based on somatic recombination of immune receptors built from immunoglobulin superfamily domains	7.61E-09	3.74E-07				
				GO:0050766	Positive regulation of phagocytosis	7.78E-09	3.74E-07				
				GO:0050851	Antigen receptor-mediated signaling pathway	7.82E-09	3.68E-07				

				GO:0030217	T cell differentiation	1.19E-08	5.46E-07				
				GO:0030098	Lymphocyte differentiation	1.48E-08	6.65E-07				
				GO:0050764	Regulation of phagocytosis	2.04E-08	8.99E-07				
				GO:0051050	Positive regulation of transport	3.47E-08	1.49E-06				
				GO:0002706	Regulation of lymphocyte mediated immunity	3.94E-08	1.67E-06				
				GO:0045807	Positive regulation of endocytosis	4.05E-08	1.68E-06				
				GO:0051130	Positive regulation of cellular component organization	4.58E-08	1.86E-06				
				GO:0002697	Regulation of immune effector process	5.12E-08	2.04E-06				
				GO:0006911	Phagocytosis, engulfment	5.31E-08	2.08E-06				
				GO:0002274	Myeloid leukocyte activation	7.77E-08	2.99E-06				
				GO:0002683	Negative regulation of immune system process	9.21E-08	3.48E-06				
				GO:0002703	Regulation of leukocyte mediated immunity	1.10E-07	4.09E-06				
				GO:0030097	Hemopoiesis	1.22E-07	4.46E-06				
				GO:0002526	Acute inflammatory response	1.55E-07	5.56E-06				
				GO:0045619	Regulation of lymphocyte differentiation	1.83E-07	6.46E-06				
				GO:0002444	Myeloid leukocyte mediated immunity	1.88E-07	6.52E-06				
				GO:0006909	Phagocytosis	2.30E-07	7.83E-06				
				GO:0048534	Hemopoietic or lymphoid organ development	2.94E-07	9.85E-06				
				GO:0007610	Behavior	4.23E-07	1.40E-05				
				GO:0002520	Immune system development	7.38E-07	2.40E-05				
				GO:0050864	Regulation of B cell activation	8.43E-07	2.70E-05				
				GO:0050727	Regulation of inflammatory response	8.43E-07	2.70E-05				
				GO:0008283	Cell proliferation	1.74E-06	5.49E-05				
				GO:0002861	Regulation of inflammatory response to antigenic stimulus	2.75E-06	8.55E-05				
				GO:0002824	Positive regulation of adaptive immune response based on somatic recombination of immune receptors built from immunoglobulin superfamily domains	3.66E-06	1.12E-04				
				GO:0002821	Positive regulation of adaptive immune response	3.66E-06	1.12E-04				
				GO:0030100	Regulation of endocytosis	3.82E-06	1.15E-04				
				GO:0002863	Positive regulation of inflammatory response to antigenic stimulus	4.02E-06	1.20E-04				
				GO:0006959	Humoral immune response	4.61E-06	1.35E-04				
				GO:0048585	Negative regulation of response to stimulus	5.96E-06	1.72E-04				

				GO:0045582	Positive regulation of T cell differentiation	6.21E-06	1.77E-04				
				GO:0032944	Regulation of mononuclear cell proliferation	7.46E-06	2.10E-04				
				GO:0050670	Regulation of lymphocyte proliferation	7.46E-06	2.10E-04				
				GO:0002883	Regulation of hypersensitivity	7.83E-06	2.18E-04				
				GO:0043368	Positive T cell selection	7.83E-06	2.18E-04				
				GO:0002864	Regulation of acute inflammatory response to antigenic stimulus	7.83E-06	2.18E-04				
				GO:0019221	Cytokine-mediated signaling pathway	7.92E-06	2.17E-04				
				GO:0009617	Response to bacterium	8.27E-06	2.24E-04				
				GO:0070663	Regulation of leukocyte proliferation	9.76E-06	2.61E-04				
				GO:0045621	Positive regulation of lymphocyte differentiation	1.01E-05	2.67E-04				
				GO:0002891	Positive regulation of immunoglobulin mediated immune response	1.40E-05	3.65E-04				
				GO:0002714	Positive regulation of B cell mediated immunity	1.40E-05	3.65E-04				
				GO:0001909	Leukocyte mediated cytotoxicity	1.40E-05	3.65E-04				
				GO:0042129	Regulation of T cell proliferation	1.54E-05	3.96E-04				
				GO:0001810	Regulation of type I hypersensitivity	1.57E-05	4.00E-04				
				GO:0045580	Regulation of T cell differentiation	1.70E-05	4.28E-04				
				GO:0001819	Positive regulation of cytokine production	2.10E-05	5.21E-04				
				GO:0051094	Positive regulation of developmental process	2.10E-05	5.17E-04				
				GO:0045058	T cell selection	3.01E-05	7.30E-04				
				GO:0042742	Defense response to bacterium	3.15E-05	7.57E-04				
				GO:0051250	Negative regulation of lymphocyte activation	3.39E-05	8.04E-04				
				GO:0002866	Positive regulation of acute inflammatory response to antigenic stimulus	3.57E-05	8.39E-04				
				GO:0002885	Positive regulation of hypersensitivity	3.57E-05	8.39E-04				
				GO:0002707	Negative regulation of lymphocyte mediated immunity	3.69E-05	8.56E-04				
				GO:0002704	Negative regulation of leukocyte mediated immunity	3.69E-05	8.56E-04				
				GO:0050854	Regulation of antigen receptor-mediated signaling pathway	3.69E-05	8.56E-04				
				GO:0002366	Leukocyte activation during immune response	3.90E-05	8.96E-04				
				GO:0002263	Cell activation during immune response	3.90E-05	8.96E-04				
				GO:0002695	Negative regulation of leukocyte activation	3.98E-05	9.04E-04				
				GO:0050866	Negative regulation of cell activation	3.98E-05	9.04E-04				

				GO:0050852	T cell receptor signaling pathway	4.09E-05	9.20E-04				
				GO:0032675	Regulation of interleukin-6 production	4.90E-05	1.08E-03				
				GO:0060627	Regulation of vesicle-mediated transport	5.26E-05	1.15E-03				
				GO:0045576	Mast cell activation	5.58E-05	1.21E-03				
				GO:0050853	B cell receptor signaling pathway	5.58E-05	1.21E-03				
				GO:0002675	Positive regulation of acute inflammatory response	6.96E-05	1.50E-03				
				GO:0045059	Positive thymic T cell selection	6.96E-05	1.50E-03				
				GO:0045061	Thymic T cell selection	8.15E-05	1.73E-03				
				GO:0001906	Cell killing	8.15E-05	1.73E-03				
				GO:0002673	Regulation of acute inflammatory response	8.15E-05	1.73E-03				
				GO:0032101	Regulation of response to external stimulus	8.58E-05	1.81E-03				
				GO:0032945	Negative regulation of mononuclear cell proliferation	9.21E-05	1.92E-03				
				GO:0070664	Negative regulation of leukocyte proliferation	9.21E-05	1.92E-03				
				GO:0050672	Negative regulation of lymphocyte proliferation	9.21E-05	1.92E-03				
				GO:0002708	Positive regulation of lymphocyte mediated immunity	1.12E-04	2.31E-03				
				GO:0002705	Positive regulation of leukocyte mediated immunity	1.12E-04	2.31E-03				
				GO:0010324	Membrane invagination	1.13E-04	2.32E-03				
				GO:0006897	Endocytosis	1.13E-04	2.32E-03				
				GO:0032680	Regulation of tumor necrosis factor production	1.51E-04	3.07E-03				
				GO:0009620	Response to fungus	1.59E-04	3.19E-03				
				GO:0042035	Regulation of cytokine biosynthetic process	1.87E-04	3.71E-03				
				GO:0042130	Negative regulation of T cell proliferation	1.89E-04	3.73E-03				
				GO:0016044	Membrane organization	1.93E-04	3.77E-03				
				GO:0043383	Negative T cell selection	1.98E-04	3.83E-03				
				GO:0042098	T cell proliferation	2.34E-04	4.49E-03				
				GO:0031349	Positive regulation of defense response	2.51E-04	4.77E-03				
				GO:0002698	Negative regulation of immune effector process	2.83E-04	5.33E-03				
				GO:0031348	Negative regulation of defense response	2.87E-04	5.36E-03				
				GO:0006910	Phagocytosis, recognition	3.04E-04	5.61E-03				
				GO:0006968	Cellular defense response	3.04E-04	5.61E-03				
				GO:0002699	Positive regulation of immune effector process	3.16E-04	5.79E-03				
				GO:0050868	Negative regulation of T cell activation	3.16E-04	5.79E-03				
				GO:0001812	Positive regulation of type I hypersensitivity	3.25E-04	5.91E-03				

				GO:0002700	Regulation of production of molecular mediator of immune response	3.49E-04	6.29E-03				
				GO:0050729	Positive regulation of inflammatory response	3.68E-04	6.56E-03				
				GO:0046651	Lymphocyte proliferation	3.69E-04	6.53E-03				
				GO:0009615	Response to virus	3.76E-04	6.60E-03				
				GO:0070661	Leukocyte proliferation	4.29E-04	7.46E-03				
				GO:0032943	Mononuclear cell proliferation	4.29E-04	7.46E-03				
				GO:0002886	Regulation of myeloid leukocyte mediated immunity	4.44E-04	7.66E-03				
				GO:0022610	Biological adhesion	4.79E-04	8.20E-03				
				GO:0007155	Cell adhesion	4.79E-04	8.20E-03				
				GO:0002455	Humoral immune response mediated by circulating immunoglobulin	5.05E-04	8.57E-03				
				GO:0045597	Positive regulation of cell differentiation	5.61E-04	9.43E-03				
				GO:0050728	Negative regulation of inflammatory response	5.92E-04	9.88E-03				

Neighboring area

Pattern 1				Pattern 2				Pattern 3			
**ID**	**Name**	**p-value**	**FDR**	**ID**	**Name**	**p-value**	**FDR**	**ID**	**Name**	**p-value**	**FDR**
GO:0006355	Regulation of transcription, DNA-dependent	4.16E-08	1.56E-04	GO:0006955	Immune response	2.00E-33	2.16E-30	NS	NS	NS	NS
GO:0051252	Regulation of RNA metabolic process	1.03E-07	1.94E-04	GO:0006954	Inflammatory response	2.39E-14	1.29E-11				
				GO:0006952	Defense response	1.23E-13	4.42E-11				
				GO:0009611	Response to wounding	1.63E-11	4.40E-09				
				GO:0042330	Taxis	2.75E-08	5.94E-06				
				GO:0006935	Chemotaxis	2.75E-08	5.94E-06				
				GO:0009615	Response to virus	9.79E-06	1.75E-03				
				GO:0002526	Acute inflammatory response	2.03E-05	3.12E-03				
				GO:0007626	Locomotory behavior	4.72E-05	6.34E-03				
				GO:0002684	Positive regulation of immune system process	6.48E-05	7.74E-03				

* p-values were calculated using Fischer's test.

** FDR corrections were calculated using the Benjamini-Hochberg procedure.
