# Supplementary material for: Focal exposure of limited lung volumes to high-dose irradiation down-regulated organ development-related functions and up-regulated the immune response in mouse pulmonary tissues
Source: BMC Genet. 2016 Jan 27;17:29. doi: 10.1186/s12863-016-0338-9 (PMC4729165; doi:10.1186/s12863-016-0338-9)
Supplement: Additional file 5: — Tree distribution of GO terms altered by focal exposure to high-dose radiation. Tree structures composed of non-redundant GO terms were constructed using the REIVGO program from all enriched GO terms (FDR <0.01) in focally irradiated regions and non-irradiated neighboring lung regions. In each tree structure, closely related terms are presented in the same color. The size of each GO term is proportional to the level of statistical significance. (PDF 238 kb) [file 12863_2016_338_MOESM5_ESM.pdf]

Additional file 5

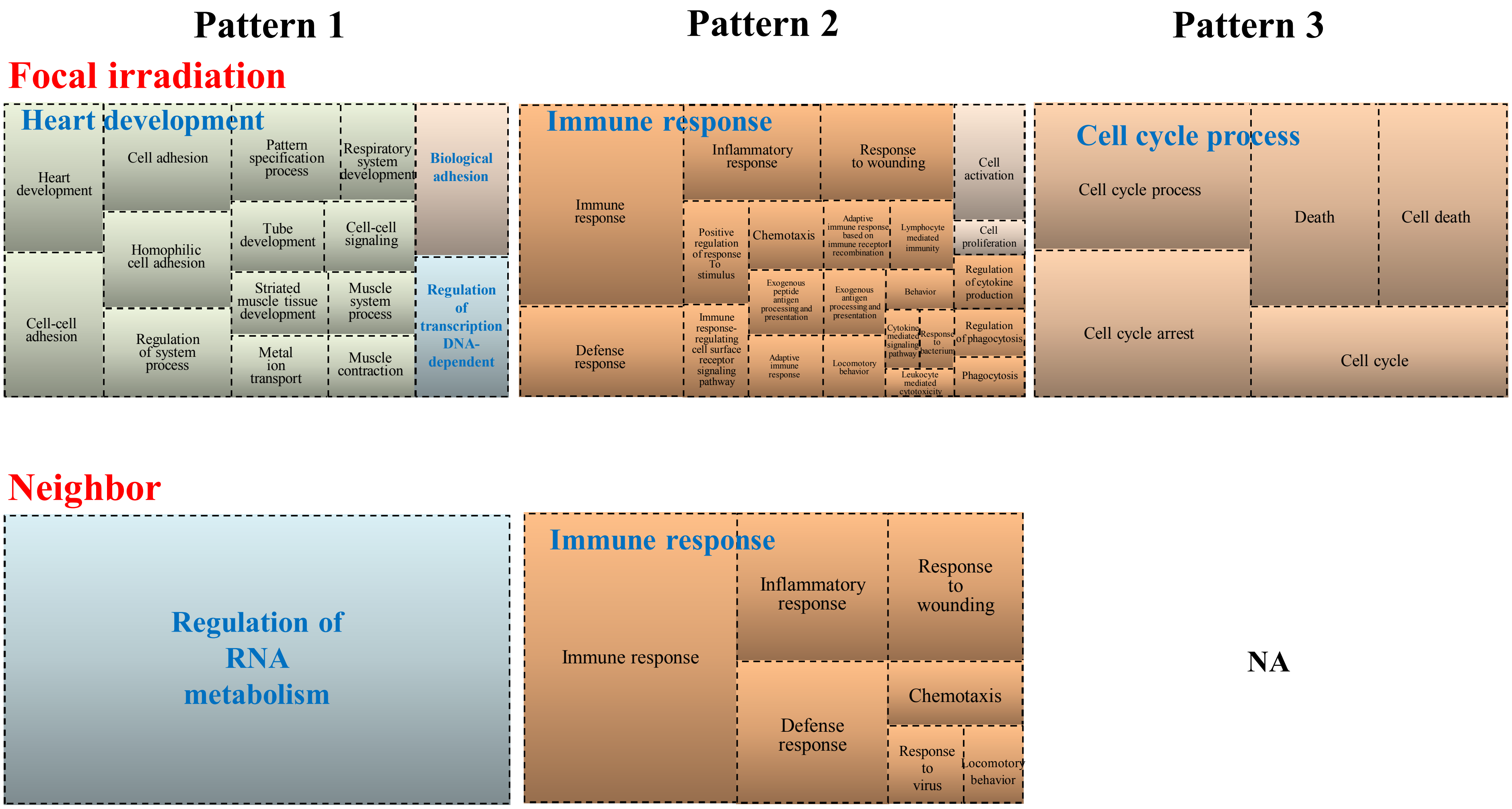

Additional file 5. Tree distribution of GO terms altered by focal exposure to high-dose radiation. Tree structures composed of non-redundant GO terms were constructed using the REIVGO program from all enriched GO terms (FDR<0.01) in focally irradiated regions and non-irradiated neighboring lung regions. In each tree structure, closely related terms are presented in the same color. The size of each GO term is proportional to the level of statistical significance.
