# Supplementary material for: Focal exposure of limited lung volumes to high-dose irradiation down-regulated organ development-related functions and up-regulated the immune response in mouse pulmonary tissues
Source: BMC Genet. 2016 Jan 27;17:29. doi: 10.1186/s12863-016-0338-9 (PMC4729165; doi:10.1186/s12863-016-0338-9)
Supplement: Additional file 6: — GO terms altered by focal exposure to 90 Gy and diffused exposure to 20 Gy. Significantly enriched non-redundant GO terms at each time point were temporally distributed. The columns represent individual samples, while the rows represent statistically significant GO terms (FDR <0.01). The positions of the organ development-related terms and immune-related terms are indicated as bars. (PDF 121 kb) [file 12863_2016_338_MOESM6_ESM.pdf]

Additional file 6

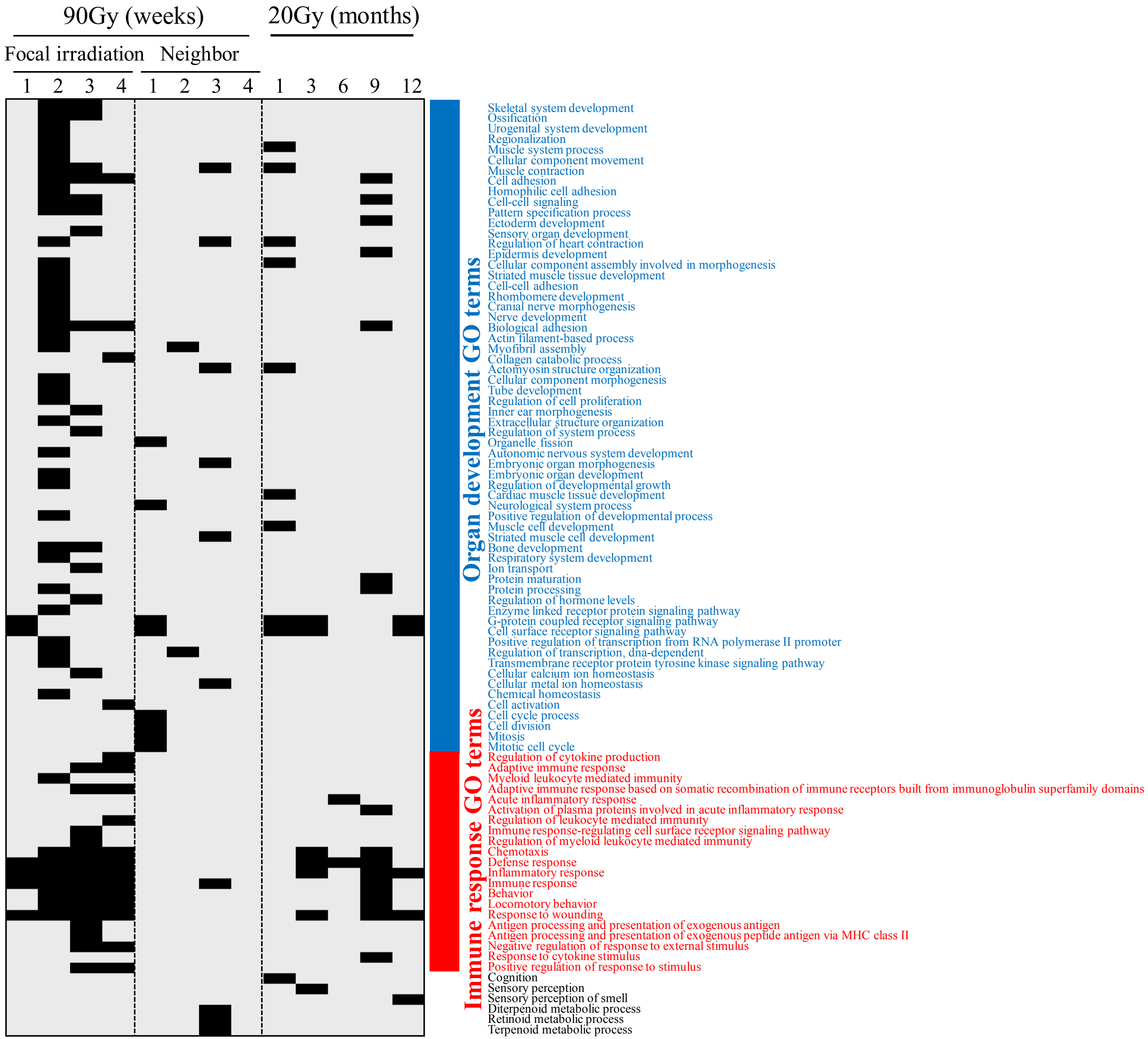

Additional file 6. GO terms altered by focal exposure to 90 Gy and diffused exposure to 20 Gy. Significantly enriched non-redundant GO terms at each time point were temporally distributed. The columns represent individual samples, while the rows represent statistically significant GO terms (FDR<0.01). The positions of the organ development-related terms and immune-related terms are indicated as bars.
