# Supplementary material for: Focal exposure of limited lung volumes to high-dose irradiation down-regulated organ development-related functions and up-regulated the immune response in mouse pulmonary tissues
Source: BMC Genet. 2016 Jan 27;17:29. doi: 10.1186/s12863-016-0338-9 (PMC4729165; doi:10.1186/s12863-016-0338-9)
Supplement: Additional file 7: — GO enrichment analysis in lung exposed to low-dosage radiation of 20 Gy. (PDF 195 kb) [file 12863_2016_338_MOESM7_ESM.pdf]

Additional file 7. GO enrichment analysis in lung exposed to low-dosage radiation of 20 Gy

Low-dosage (20Gy) irradiation

Down-pattern				Up-pattern			
**ID**	**Name**	**p-value***	**FDR****	**ID**	**Name**	**p-value**	**FDR**
GO:0007507	Heart development	3.33E-08	2.55E-05	GO:0006955	Immune response	2.64E-07	2.07E-04
GO:0003012	Muscle system process	5.58E-08	2.14E-05	GO:0006954	Inflammatory response	1.31E-06	5.14E-04
GO:0006936	Muscle contraction	4.34E-07	1.11E-04	GO:0009611	Response to wounding	7.67E-05	1.98E-03
GO:0048738	Cardiac muscle tissue development	4.34E-07	1.11E-04				
GO:0055001	Muscle cell development	7.09E-07	1.36E-04				
GO:0007517	Muscle organ development	1.49E-06	2.29E-04				
GO:0003007	Heart morphogenesis	2.09E-06	2.67E-04				
GO:0007512	Adult heart development	3.61E-06	3.96E-04				
GO:0014706	Striated muscle tissue development	8.11E-06	7.78E-04				
GO:0051146	Striated muscle cell differentiation	9.97E-06	8.50E-04				
GO:0060537	Muscle tissue development	1.32E-05	1.01E-03				
GO:0044057	Regulation of system process	2.55E-05	1.77E-03				
GO:0042692	Muscle cell differentiation	5.48E-05	3.50E-03				

* p-values were calculated using Fischer’s test.

** FDR corrections were calculated using the Benjamini-Hochberg procedure.
