# Supplementary material for: Focal exposure of limited lung volumes to high-dose irradiation down-regulated organ development-related functions and up-regulated the immune response in mouse pulmonary tissues
Source: BMC Genet. 2016 Jan 27;17:29. doi: 10.1186/s12863-016-0338-9 (PMC4729165; doi:10.1186/s12863-016-0338-9)
Supplement: Additional file 8: — Top five pathways enriched in Pattern 1, Pattern 2, and Pattern 3 in focally irradiated regions and non-irradiated neighboring lung regions. The position of each gene in the pathways is colored red for the focally irradiated regions, blue for the non-irradiated neighboring lung regions, and yellow for both regions. The image of each pathway was adopted from KEGG homepage (http://www.kegg.jp). (PDF 656 kb) [file 12863_2016_338_MOESM8_ESM.pdf]

Additional file 8

Pattern 1

Dilated cardiomyopathy

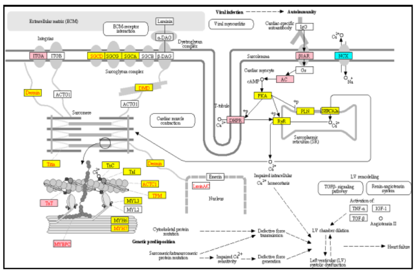

Hypertrophic cardiomyopathy

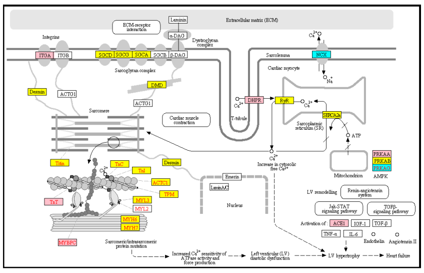

Arrhythmogenic right ventricular cardiomyopathy

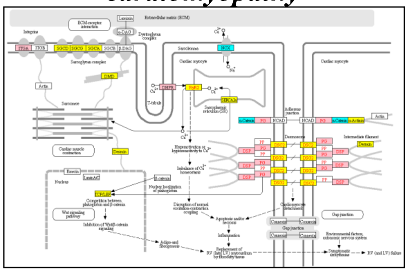

Calcium signaling pathway

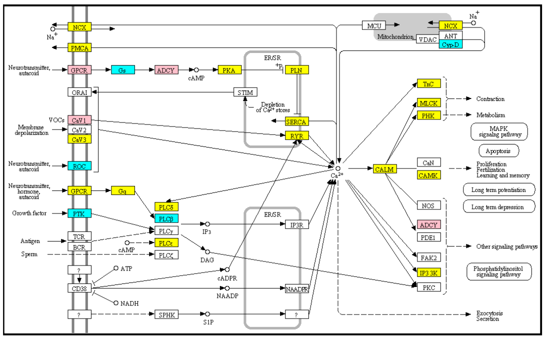

Drug metabolism

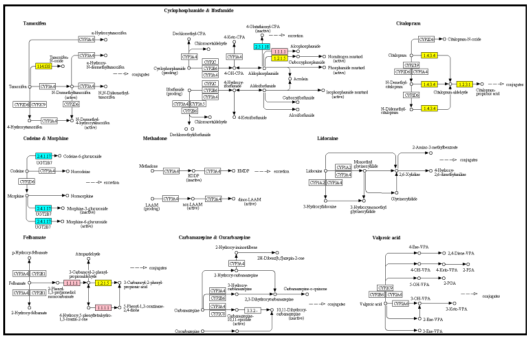

Pattern 2

Cytokine-cytokine receptor interaction

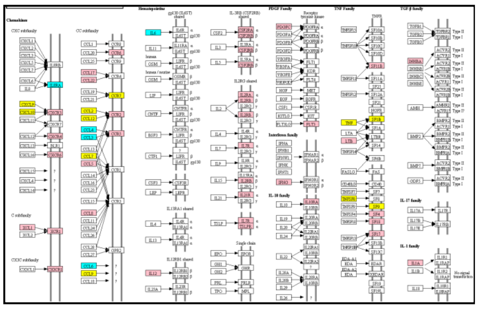

Systemic lupus erythematosus

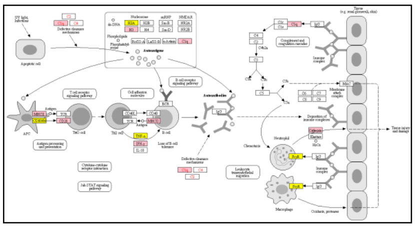

Chemokine signaling pathway

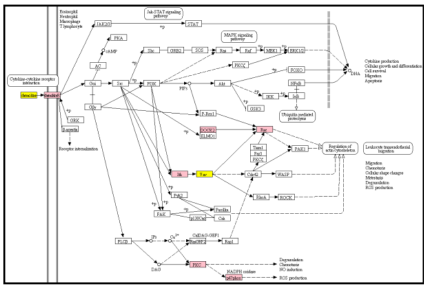

Allograft rejection

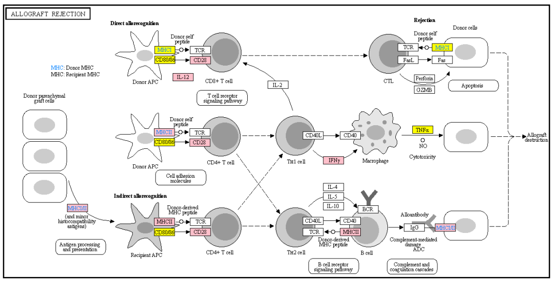

Graft-versus-host disease

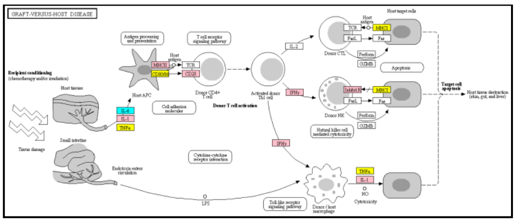

Pattern 3

p53 signaling pathway

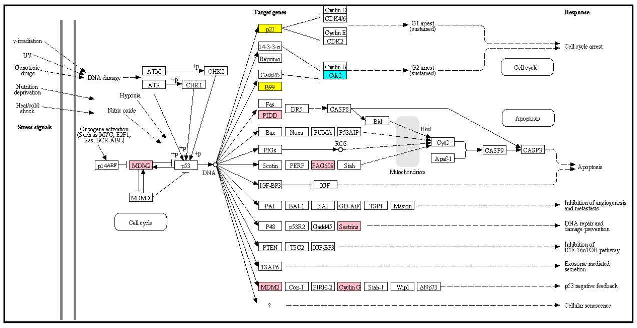

Additional file 8. Top five pathways enriched in Pattern 1, Pattern 2, and Pattern 3 in focally irradiated regions and non-irradiated neighboring lung regions. The position of each gene in the pathways is colored red for the focally irradiated regions, blue for the non-irradiated neighboring lung regions, and yellow for both regions. The image of each pathway was adopted from KEGG homepage (http://www.kegg.jp).
