# Supplementary material for: Focal exposure of limited lung volumes to high-dose irradiation down-regulated organ development-related functions and up-regulated the immune response in mouse pulmonary tissues
Source: BMC Genet. 2016 Jan 27;17:29. doi: 10.1186/s12863-016-0338-9 (PMC4729165; doi:10.1186/s12863-016-0338-9)
Supplement: Additional file 9: — Pathway enrichment analysis in lung exposed to low-dosage radiation of 20 Gy. (PDF 192 kb) [file 12863_2016_338_MOESM9_ESM.pdf]

Additional file 9. Pathway enrichment analysis in lung exposed to low-dosage radiation of 20 Gy

Low-dosage (20Gy) irradiation

Down-pattern				Up-pattern			
**ID**	**Name**	**p-value***	**FDR****	**ID**	**Name**	**p-value**	**FDR**
mmu04260	Cardiac muscle contraction	7.13E-08	4.71E-06	mmu04060	Cytokine-cytokine receptor interaction	4.37E-04	0.028873
mmu05410	Hypertrophic cardiomyopathy (HCM)	3.95E-05	0.001304				
mmu05414	Dilated cardiomyopathy	6.75E-05	0.001484				

* p-values were calculated using Fischer’s test.

** FDR corrections were calculated using the Benjamini-Hochberg procedure.
