# Supplementary material for: Focal exposure of limited lung volumes to high-dose irradiation down-regulated organ development-related functions and up-regulated the immune response in mouse pulmonary tissues
Source: BMC Genet. 2016 Jan 27;17:29. doi: 10.1186/s12863-016-0338-9 (PMC4729165; doi:10.1186/s12863-016-0338-9)
Supplement: Additional file 10: — Network structure of GO terms associated with modules from (A) focally-irradiated (90 Gy) regions and (B) non-irradiated neighboring lung regions. The network structure among non-redundant GO terms was constructed from all enriched GO terms (FDR <0.01) using the REIVGO program. The node size and color intensity are proportional to the hierarchical status and statistical significance of each node, respectively. The edge thickness between nodes represents the closeness of two nodes. (PDF 211 kb) [file 12863_2016_338_MOESM10_ESM.pdf]

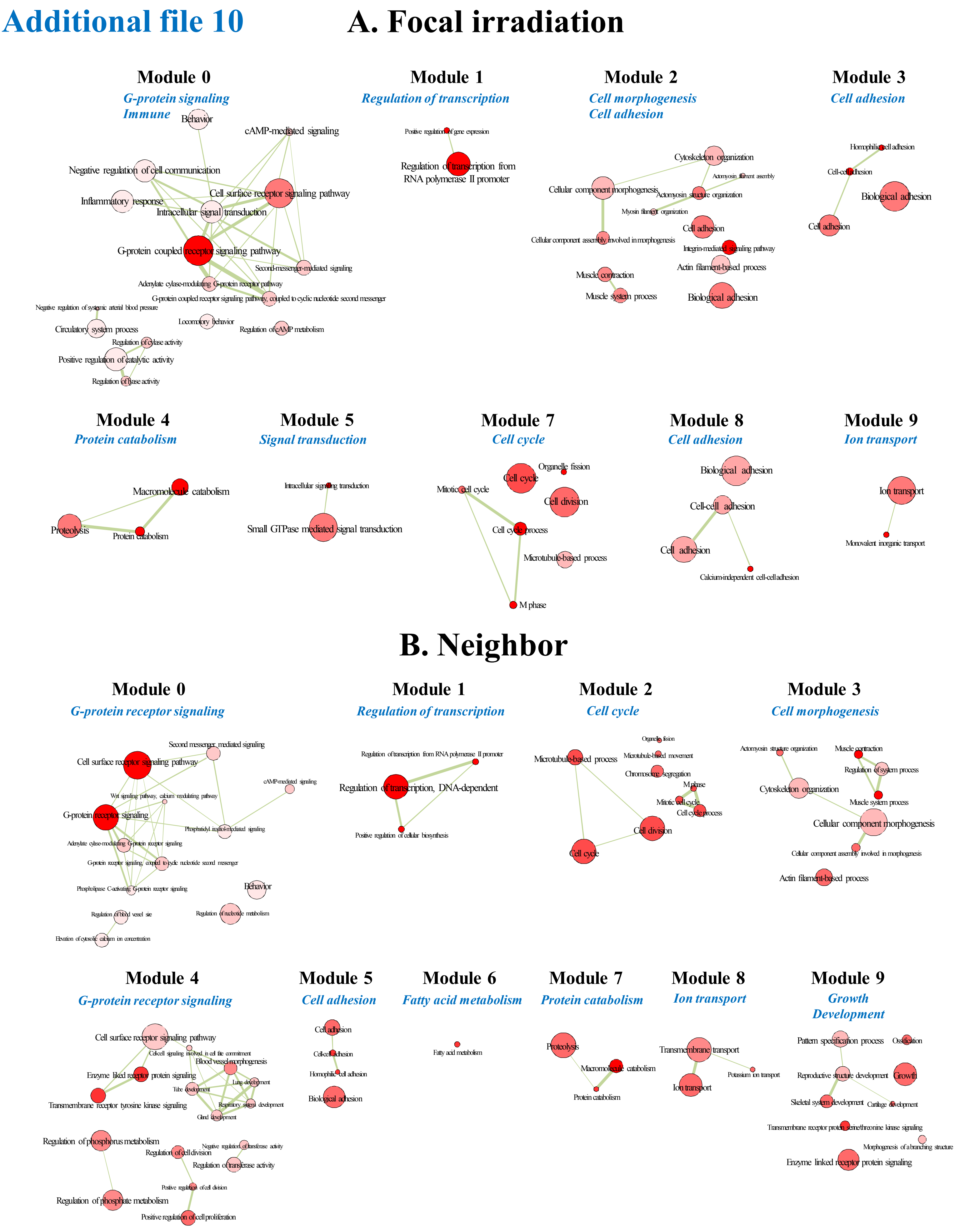

Additional file 10. Network structure of GO terms associated with modules from (A) focally-irradiated (90 Gy) regions and (B) non-irradiated neighboring lung regions. The network structure among non-redundant GO terms was constructed from all enriched GO terms (FDR<0.01) using the REIVGO program. The node size and color intensity are proportional to the hierarchical status and statistical significance of each node, respectively. The edge thickness between nodes represents the closeness of two nodes.
