# Supplementary material for: Focal exposure of limited lung volumes to high-dose irradiation down-regulated organ development-related functions and up-regulated the immune response in mouse pulmonary tissues
Source: BMC Genet. 2016 Jan 27;17:29. doi: 10.1186/s12863-016-0338-9 (PMC4729165; doi:10.1186/s12863-016-0338-9)
Supplement: Additional file 11: — Genes included in each module of network structure. (PDF 363 kb) [file 12863_2016_338_MOESM11_ESM.pdf]

Additional file 11. Genes included in each module of network structure

Focally irradiated area							Neighboring area					
**Module**	**Name**	**Degree**	**Betweenness Centrality**	**Closeness Centrality**	**Clustering Coefficient**		**Module**	**Name**	**Degree**	**Betweenness Centrality**	**Closeness Centrality**	**Clustering Coefficient**
0	GNB5	95	0.0896	0.3464	0.0788		0	GNG4	91	0.0849	0.2606	0.0991
0	GNG3	94	0.0764	0.3461	0.0824		0	GNG8	91	0.0849	0.2606	0.0991
0	GNG4	94	0.0764	0.3461	0.0824		0	GNB3	89	0.0821	0.26	0.0817
0	GNG8	94	0.0764	0.3461	0.0824		0	GNB4	88	0.0803	0.2598	0.0825
0	GNA14	38	0.0322	0.3066	0.2006		0	GNG13	88	0.0803	0.26	0.0805
0	GNAO1	35	0.1719	0.3813	0.1731		0	GNB5	82	0.0769	0.2589	0.0867
0	GNAZ	17	0.0484	0.3421	0.375		0	GNA14	44	0.0229	0.2498	0.2484
0	KCNJ5	12	0.0001	0.3015	0.6667		0	KCNJ5	12	0	0.2085	0.7121
0	KCNJ6	12	0.0001	0.3015	0.6667		0	KCNJ6	12	0	0.2085	0.7121
0	CALCRL	9	0.0001	0.2658	0.7778		0	WNT5A	11	0.0078	0.2088	0.2909
0	CHRM2	8	0.0002	0.298	0.8571		0	GABBR1	10	0	0.2082	0.7556
0	GABBR1	8	0.0003	0.2975	0.8214		0	GABBR2	10	0	0.2082	0.7556
0	NTS	8	0.0234	0.3436	0.5714		0	NTS	10	0.2088	0.2765	0.6667
0	SSTR2	8	0.0241	0.3339	0.7143		0	WNT11	10	0.0021	0.2086	0.3556
0	ADM	7	0	0.2654	0.9524		0	BDKRB1	9	0	0.2081	0.7778
0	ADORA3	7	0	0.2973	0.9524		0	DKK2	9	0.0001	0.1742	0
0	EDNRB	7	0.0002	0.2977	0.9048		0	SFRP2	9	0.0001	0.1742	0
0	GRM2	7	0	0.2973	0.9524		0	SSTR3	9	0	0.2081	0.6667
0	RAMP2	7	0.0208	0.3027	0.7143		0	SSTR4	9	0	0.2081	0.6667
0	RGS1	7	0.0012	0.3174	0.6667		0	WIF1	9	0.0001	0.1742	0
0	SSTR4	7	0	0.2973	0.9524		0	WNT10A	9	0.0018	0.2083	0.3333
0	TSHB	7	0.0219	0.343	0.5238		0	WNT16	9	0.0018	0.2083	0.3333
0	CCK	6	0	0.2703	1		0	WNT2	9	0.0018	0.2083	0.3333
0	CCKAR	6	0	0.2703	1		0	WNT6	9	0.0018	0.2083	0.3333
0	CRHR1	6	0	0.297	1		0	WNT7A	9	0.0018	0.2083	0.3333

0	CXCL10	6	0	0.2589	0.9333		0	WNT7B	9	0.0018	0.2083	0.3333
0	CXCL9	6	0	0.2589	0.9333		0	WNT9A	9	0.0018	0.2083	0.3333
0	FPR2	6	0	0.2703	1		0	CALCA	8	0.0118	0.2166	0.5714
0	GNRH1	6	0.0132	0.331	0.6667		0	EDN3	8	0	0.208	0.8214
0	GPR45	6	0	0.2963	0.9333		0	EDNRB	8	0	0.208	0.8214
0	HTR2C	6	0.0021	0.2871	0.6667		0	FPR2	8	0	0.208	0.8214
0	NTSR2	6	0	0.2845	1		0	GPR65	8	0.03	0.2239	0.5714
0	PTGER1	6	0.0021	0.2871	0.6667		0	GRM2	8	0	0.208	0.7857
0	PTGER4	6	0.0199	0.3427	0.4667		0	NTSR1	8	0	0.2373	0.8214
0	RAMP3	6	0	0.2589	1		0	NTSR2	8	0	0.2373	0.8214
0	S1PR5	6	0	0.297	1		0	PTGER1	8	0.1231	0.2137	0.5714
0	SAA1	6	0	0.2703	1		0	SAA1	8	0	0.208	0.8214
0	VIP	6	0.0139	0.3307	0.6667		0	ADCYAP1	7	0	0.2078	0.7619
0	ADRA1A	5	0	0.2701	1		0	ADCYAP1R1	7	0	0.2078	0.7619
0	ADRA1D	5	0	0.2701	1		0	ADM	7	0.0116	0.2165	0.4762
0	ADRA2B	5	0	0.2785	1		0	ADRA1A	7	0	0.2078	0.7619
0	ADRA2C	5	0	0.2785	1		0	ADRA1D	7	0	0.2078	0.7619
0	ADRB2	5	0.0019	0.2863	0.6		0	CALCR	7	0	0.2095	0.7619
0	APLN	5	0	0.2587	1		0	FPR3	7	0	0.2078	0.7619
0	APLNR	5	0	0.2587	1		0	GNAL	7	0	0.2077	0.5238
0	AVPR1A	5	0	0.2701	1		0	GPR4	7	0	0.2078	0.7619
0	CALCA	5	0	0.2587	1		0	GPR68	7	0	0.2078	0.7619
0	CALCB	5	0	0.2587	1		0	GRP	7	0	0.2078	0.7619
0	CCL19	5	0	0.2587	1		0	HCRT	7	0	0.2078	0.7619
0	CXCL1	5	0.0121	0.3304	0.6		0	HEBP1	7	0	0.2078	0.7619
0	CXCL13	5	0	0.2587	1		0	HRH3	7	0	0.2078	0.5714
0	GAST	5	0	0.2701	1		0	HRH4	7	0	0.2078	0.5714
0	GCGR	5	0	0.2701	1		0	HTR7	7	0	0.2078	0.5714
0	GPR132	5	0	0.2701	1		0	KISS1	7	0	0.2078	0.7619
0	GPR65	5	0	0.2701	1		0	LPAR5	7	0	0.2078	0.7619
0	GPR68	5	0	0.2701	1		0	MTNR1A	7	0	0.2078	0.7619
0	GPRC6A	5	0	0.2701	1		0	MTNR1B	7	0	0.2078	0.7619
0	GPSM1	5	0	0.2961	1		0	OPN4	7	0	0.2078	0.7619

0	GRP	5	0	0.2701	1		0	P2RY1	7	0	0.2078	0.7619
0	HTR1D	5	0	0.2968	1		0	P2RY6	7	0	0.2078	0.7619
0	KISS1	5	0	0.2701	1		0	PROKR1	7	0	0.2078	0.7619
0	LPAR4	5	0	0.2701	1		0	PTGER3	7	0	0.2078	0.7619
0	LTB4R2	5	0	0.2701	1		0	PTGFR	7	0	0.2078	0.7619
0	MTNR1A	5	0	0.2587	1		0	SCT	7	0	0.2078	0.7619
0	MTNR1B	5	0	0.2587	1		0	SCTR	7	0	0.2078	0.7619
0	NPFF	5	0	0.2701	1		0	TACR1	7	0	0.2078	0.7619
0	NPS	5	0	0.2701	1		0	TACR2	7	0	0.2078	0.7619
0	P2RY10	5	0	0.2701	1		0	TRH	7	0	0.2078	0.7619
0	P2RY6	5	0	0.2701	1		0	TSHB	7	0.1826	0.2758	0.4762
0	PTAFR	5	0	0.2701	1		0	UTS2R	7	0	0.2078	0.7619
0	PTGFR	5	0	0.2701	1		0	XCL1	7	0	0.2078	0.7619
0	TACR2	5	0	0.2701	1		0	ADRA2B	6	0	0.2077	0.6667
0	TSHR	5	0	0.2837	1		0	APLNR	6	0	0.2077	0.6667
0	UTS2R	5	0	0.2701	1		0	CCL19	6	0	0.2077	0.6667
0	VIPR2	5	0	0.2814	1		0	CCL25	6	0	0.2077	0.6667
0	XCL1	5	0	0.2701	1		0	CNR2	6	0	0.2077	0.6667
0	ADRB3	4	0	0.2585	1		0	CRHR1	6	0	0.2077	0.6667
0	C3AR1	4	0	0.2585	1		0	CRHR2	6	0	0.2077	0.6667
0	CCL25	4	0	0.2585	1		0	GALR3	6	0	0.2077	0.6667
0	CNR2	4	0	0.2585	1		0	GHRH	6	0	0.2077	0.6667
0	CRHR2	4	0	0.2585	1		0	GLP1R	6	0	0.2077	0.6667
0	GLP1R	4	0	0.2585	1		0	GPBAR1	6	0	0.2077	0.6667
0	GPBAR1	4	0	0.2585	1		0	GPR18	6	0	0.2077	0.6667
0	HEBP1	4	0	0.2585	1		0	INSL5	6	0	0.2077	0.6667
0	HRH3	4	0	0.2585	1		0	NPB	6	0	0.2077	0.6667
0	HRH4	4	0	0.2585	1		0	NPW	6	0	0.2077	0.6667
0	HTR7	4	0	0.2585	1		0	OPN1MW	6	0	0.2077	0.6667
0	INSL5	4	0	0.2585	1		0	OPN3	6	0	0.2077	0.6667
0	KCNJ10	4	0	0.2578	1		0	PPY	6	0	0.2077	0.6667
0	NPB	4	0	0.2585	1		0	PTGIR	6	0	0.2077	0.6667
0	NPW	4	0	0.2585	1		0	PTH1R	6	0	0.2077	0.6667

0	PPY	4	0	0.2585	1		0	PYY	6	0	0.2077	0.6667
0	PTGER2	4	0	0.2585	1		0	S1PR4	6	0	0.2077	0.6667
0	PTGIR	4	0	0.2585	1		0	VIPR1	6	0	0.2077	0.6667
0	PTH1R	4	0	0.2585	1		0	VIPR2	6	0	0.2077	0.6667
0	RGS9	4	0.0014	0.2963	0.6667		0	KCNJ2	5	0	0.2075	1
0	PDE6G	3	0	0.2578	1		0	CCL11	2	0.1181	0.1801	0
0	RGS9BP	3	0	0.2578	1		0	PSD	1	0	0.2058	0
0	PLA2G4D	2	0	0.2768	1		0	VANGL2	1	0	0.1728	0
0	RGS16	2	0	0.2764	1		1	EGR1	27	0.267	0.26	0.0085
0	PSD	1	0	0.2575	0		1	FOS	24	0.52	0.3009	0.0109
0	RGS10	1	0	0.2762	0		1	MEF2C	12	0.0609	0.2383	0.0303
0	RGS11	1	0	0.2762	0		1	TRAF6	11	0.0947	0.2384	0.0364
0	RGS12	1	0	0.2762	0		1	IL1RN	5	0.0192	0.2488	0.3
0	RGS5	1	0	0.2762	0		1	JUNB	5	0.016	0.2511	0.4
0	RGS6	1	0	0.2762	0		1	ANKRD1	3	0.3186	0.2734	0
1	JUN	44	0.2266	0.3907	0.0317		1	ASCL1	3	0.0025	0.2194	0.6667
1	FOS	28	0.0824	0.3665	0.0476		1	DUSP1	3	0.0342	0.2444	0.3333
1	EGR1	20	0.0701	0.3417	0.0526		1	IL1R2	3	0.0001	0.2103	0.6667
1	GATA2	17	0.0682	0.3396	0.0368		1	IL1RAP	3	0.0001	0.2103	0.6667
1	MYC	17	0.0557	0.3172	0.0368		1	MEF2D	3	0.026	0.2467	0.3333
1	PPARA	13	0.0487	0.2867	0.0897		1	AFAP1L2	2	0.0205	0.2465	0
1	CCL4	6	0.0111	0.3166	0.1333		1	FOSB	2	0	0.2368	1
1	CPT1B	4	0	0.2233	0.5		1	ABCA2	1	0	0.2065	0
1	CPT1C	4	0	0.2233	0.5		1	ABLIM2	1	0	0.2065	0
1	ACSBG1	3	0	0.2232	0.6667		1	AHI1	1	0	0.2065	0
1	ACSL1	3	0	0.2232	0.6667		1	ARID4A	1	0	0.2315	0
1	ACSL3	3	0	0.2232	0.6667		1	BATF3	1	0	0.2315	0
1	CD69	3	0.0003	0.2945	0.6667		1	BCL9L	1	0	0.2065	0
1	EGR2	3	0.0003	0.2945	0.6667		1	BLOC1S3	1	0	0.2315	0
1	TIMP1	3	0.0003	0.2945	0.6667		1	BST2	1	0	0.2315	0
1	AFAP1L2	2	0	0.292	1		1	CITED4	1	0	0.2065	0
1	ARNTL	2	0.001	0.2727	0		1	CRYM	1	0	0.2065	0
1	CHGA	2	0	0.292	1		1	CSRNP1	1	0	0.2315	0

1	FABP1	2	0.0008	0.245	0		1	FIGNL1	1	0	0.2065	0
1	FOSB	2	0	0.29	1		1	FOXA3	1	0	0.2065	0
1	FOSL1	2	0	0.29	1		1	GSDMA	1	0	0.1925	0
1	FOXL1	2	0	0.2698	1		1	GUCY2C	1	0	0.2315	0
1	HYAL1	2	0	0.2698	1		1	HSF4	1	0	0.2065	0
1	LTBP2	2	0	0.29	1		1	ING2	1	0	0.2065	0
1	PDE4A	2	0.0005	0.2589	0		1	IRX4	1	0	0.1925	0
1	RNF128	2	0	0.29	1		1	JHDM1D	1	0	0.2065	0
1	TFPI2	2	0	0.292	1		1	KCNK3	1	0	0.2065	0
1	TRIM33	2	0.0005	0.2589	0		1	KRT13	1	0	0.1925	0
1	USP9X	2	0	0.2841	1		1	KRT16	1	0	0.2315	0
1	ASPH	1	0	0.2812	0		1	LY9	1	0	0.1925	0
1	BLOC1S3	1	0	0.2684	0		1	MAFB	1	0	0.2315	0
1	BNC1	1	0	0.2537	0		1	MALT1	1	0	0.1926	0
1	BST2	1	0	0.2684	0		1	MAMSTR	1	0	0.2065	0
1	CD7	1	0	0.2537	0		1	MAZ	1	0	0.2315	0
1	CD93	1	0	0.2537	0		1	NLRX1	1	0	0.1926	0
1	CGREF1	1	0	0.2409	0		1	NQO1	1	0	0.2315	0
1	CRYM	1	0	0.2549	0		1	PDE4A	1	0	0.2065	0
1	CSRNP1	1	0	0.2684	0		1	PELI3	1	0	0.1926	0
1	DLK1	1	0	0.2537	0		1	PERP	1	0	0.1925	0
1	DNAJC2	1	0	0.2409	0		1	POR	1	0	0.2065	0
1	DOCK9	1	0	0.2537	0		1	PTGDS	1	0	0.2315	0
1	ESM1	1	0	0.2812	0		1	RNF128	1	0	0.2315	0
1	FAIM3	1	0	0.2409	0		1	SDCBP2	1	0	0.2315	0
1	FIGNL1	1	0	0.2549	0		1	SOX18	1	0	0.1925	0
1	HAMP	1	0	0.2409	0		1	TLR8	1	0	0.1926	0
1	HMGCS2	1	0	0.2229	0		1	TNFRSF13B	1	0	0.1926	0
1	HSF4	1	0	0.2549	0		1	TNFRSF17	1	0	0.1926	0
1	LBP	1	0	0.2812	0		1	TNRC6B	1	0	0.2315	0
1	LENEP	1	0	0.2812	0		1	TRIM33	1	0	0.2065	0
1	LIPA	1	0	0.2229	0		1	TWIST2	1	0	0.1925	0
1	LRRC32	1	0	0.2812	0		1	ZKSCAN2	1	0	0.2315	0

1	MACROD1	1	0	0.2409	0		2	PLK1	30	0.093	0.2244	0.2966
1	MAMSTR	1	0	0.2549	0		2	KIF2C	18	0.0094	0.2203	0.719
1	MSR1	1	0	0.2684	0		2	PMF1	17	0.1597	0.2638	0.7721
1	MTHFD2	1	0	0.2409	0		2	CDCA5	15	0	0.2157	1
1	NNMT	1	0	0.2812	0		2	CDCA8	15	0	0.2157	1
1	NOL3	1	0	0.2812	0		2	CENPA	15	0	0.2157	1
1	NQO1	1	0	0.2684	0		2	CENPH	15	0	0.2157	1
1	PITX3	1	0	0.2812	0		2	CENPO	15	0	0.2157	1
1	PKIA	1	0	0.2537	0		2	CENPT	15	0	0.2157	1
1	PKNOX2	1	0	0.2684	0		2	DYNLL1	15	0.0109	0.1889	0.3333
1	PLTP	1	0	0.2229	0		2	ESPL1	15	0	0.2157	1
1	POR	1	0	0.2549	0		2	KNTC1	15	0	0.2157	1
1	PROCR	1	0	0.2537	0		2	NUF2	15	0	0.2157	1
1	PSAP	1	0	0.2537	0		2	SGOL1	15	0	0.2157	1
1	PSORS1C2	1	0	0.2812	0		2	SGOL2	15	0	0.2157	1
1	PUS1	1	0	0.2409	0		2	SKA1	15	0	0.2157	1
1	RCAN1	1	0	0.2812	0		2	SPC24	15	0	0.2157	1
1	RNASE4	1	0	0.2684	0		2	DCTN2	14	0.0503	0.2245	0.3736
1	SLC6A2	1	0	0.2812	0		2	DYNC1I1	8	0.0024	0.1857	0.4643
1	SP5	1	0	0.2537	0		2	CEP250	7	0	0.1881	1
1	SPRR1A	1	0	0.2812	0		2	CEP72	7	0	0.1881	1
1	SUPT7L	1	0	0.2409	0		2	HAUS2	7	0	0.1881	1
1	TOX2	1	0	0.2812	0		2	NINL	7	0	0.1881	1
1	UCP1	1	0	0.2229	0		2	TUBGCP5	7	0	0.1881	1
2	ACTN2	30	0.0428	0.3061	0.3586		2	KIF11	4	0	0.1839	1
2	ACTN3	29	0.0375	0.3056	0.3842		2	KIF5A	4	0	0.1839	1
2	PTK2	26	0.1236	0.3586	0.2954		2	KIF22	3	0	0.1838	1
2	ITGA8	18	0.0054	0.3032	0.6209		2	KIF3B	3	0	0.1838	1
2	TPM2	18	0.0015	0.2381	0.6797		2	TUBB6	2	0	0.159	1
2	ITGA1	16	0.0167	0.2709	0.3583		2	AAK1	1	0	0.1833	0
2	ITGBL1	15	0.0013	0.3025	0.781		2	ASPM	1	0	0.1833	0
2	LIMS2	15	0.0013	0.3025	0.781		2	PHLDA3	1	0	0.1589	0
2	MYL3	15	0	0.237	0.9429		2	RECQL5	1	0	0.1833	0

2	MYL4	15	0	0.237	0.9429		2	SIK2	1	0	0.1833	0
2	NEB	15	0	0.237	0.9429		2	SMC2	1	0	0.1833	0
2	PARVB	15	0.0013	0.3025	0.781		2	TPPP3	1	0	0.1833	0
2	TCAP	15	0	0.237	0.9429		3	TTN	25	0.3084	0.2335	0.55
2	TMOD1	15	0	0.237	0.9429		3	ACTN2	23	0.2098	0.2001	0.6522
2	TNNI2	15	0	0.237	0.9429		3	DES	19	0	0.1956	0.9649
2	COL13A1	14	0.0013	0.3022	0.7582		3	MYH6	19	0	0.1956	0.9649
2	COL8A2	14	0.0013	0.3022	0.7582		3	MYL1	19	0	0.1956	0.9649
2	MYBPC1	14	0	0.2369	0.9451		3	MYL3	19	0	0.1956	0.9649
2	MYBPC3	14	0	0.2369	0.9451		3	MYL4	19	0	0.1956	0.9649
2	TNNC1	14	0	0.2369	0.9451		3	NEB	19	0	0.1956	0.9649
2	TNNC2	14	0	0.2369	0.9451		3	TCAP	19	0	0.1956	0.9649
2	ITGA2B	13	0.0149	0.2968	0.4872		3	TMOD1	19	0	0.1956	0.9649
2	TNNT1	13	0	0.2367	0.9615		3	TNNI3	19	0.0057	0.1956	0.8655
2	TNNT2	13	0	0.2367	0.9615		3	TPM2	19	0	0.1956	0.9649
2	TNNT3	13	0	0.2367	0.9615		3	MYBPC1	18	0	0.1955	0.9673
2	ITGA10	12	0.0012	0.2696	0.5758		3	MYBPC3	18	0	0.1955	0.9673
2	ITGA11	12	0.0012	0.2696	0.5758		3	TNNC1	18	0	0.1955	0.9673
2	ITGAE	10	0.0271	0.3237	0.7556		3	TNNC2	18	0	0.1955	0.9673
2	ITGAX	10	0.0252	0.3215	0.7556		3	TNNI2	18	0	0.1954	0.9673
2	ITGAD	9	0	0.269	0.9444		3	TNNT1	17	0	0.1954	0.9779
2	CHAD	6	0.0002	0.274	0.6		3	TNNT2	17	0	0.1954	0.9779
2	PARVG	5	0.0002	0.2403	0.4		3	TNNT3	17	0	0.1954	0.9779
2	TLN2	5	0.0002	0.2403	0.4		3	ANKRD2	1	0	0.1894	0
2	LMOD1	3	0	0.2151	1		3	CAPN3	1	0	0.1894	0
2	MAGI1	3	0.0114	0.2961	0		3	LDB3	1	0	0.1668	0
2	MYH11	3	0	0.2151	1		3	MAGI1	1	0	0.1668	0
2	EDIL3	1	0	0.2641	0		3	MYOM1	1	0	0.1894	0
2	LDB3	1	0	0.2345	0		3	MYOM2	1	0	0.1894	0
2	MAPK8IP3	1	0	0.2641	0		3	OBSL1	1	0	0.1894	0
2	PTP4A3	1	0	0.2133	0		3	PKD2	1	0	0.1637	0
2	PTRH2	1	0	0.2641	0		4	EGFR	22	0.2161	0.2694	0.1299
2	UNC5C	1	0	0.2641	0		4	SOS1	20	0.0307	0.2158	0.2053

3	CTNNB1	51	0.3046	0.3903	0.1945		4	FGF1	8	0.0017	0.2142	0.8929
3	CTNNAL1	24	0.0003	0.2858	0.8442		4	FGF10	8	0.0017	0.2142	0.8929
3	PCDHB11	23	0.0111	0.3112	0.917		4	FGF18	8	0.0017	0.2142	0.8929
3	CDH13	22	0	0.2854	1		4	FGF6	8	0.0017	0.2142	0.8929
3	CDH16	22	0	0.2854	1		4	FGF9	8	0.0017	0.2142	0.8929
3	CDH22	22	0	0.2854	1		4	FGFR1	7	0	0.1781	0.7619
3	CELSR2	22	0	0.2854	1		4	FLT4	6	0	0.178	1
3	DCHS1	22	0	0.2854	1		4	BTC	5	0	0.2138	1
3	PCDH1	22	0	0.2854	1		4	EREG	5	0	0.2138	1
3	PCDH20	22	0	0.2854	1		4	NRG2	5	0	0.2138	1
3	PCDHAC2	22	0	0.2854	1		4	NRG4	5	0	0.2138	1
3	PCDHB1	22	0	0.2854	1		4	SPRY4	3	0.0008	0.2135	0.6667
3	PCDHB10	22	0	0.2854	1		4	AREG	2	0	0.23	1
3	PCDHB13	22	0	0.2854	1		4	NF1	2	0	0.2134	1
3	PCDHB14	22	0	0.2854	1		4	RASAL2	2	0	0.2134	1
3	PCDHB15	22	0	0.2854	1		4	IQSEC1	1	0	0.2124	0
3	PCDHB16	22	0	0.2854	1		4	KLRK1	1	0	0.1776	0
3	PCDHB18	22	0	0.2854	1		4	NDN	1	0	0.2124	0
3	PCDHB3	22	0	0.2854	1		4	ROS1	1	0	0.2124	0
3	PCDHB4	22	0	0.2854	1		4	SH3BP5	1	0	0.1776	0
3	PCDHB6	22	0	0.2854	1		4	SPRED2	1	0	0.1776	0
3	PCDHGA1	22	0	0.2854	1		4	SPRED3	1	0	0.1776	0
3	PCDHGC5	22	0	0.2854	1		4	TREM2	1	0	0.1776	0
3	FRAT1	5	0.0006	0.313	0.6		4	UCHL1	1	0	0.2124	0
3	ARR3	4	0.0283	0.3234	0.5		5	CTNNA2	23	0.1242	0.1706	0.6759
3	SAG	3	0	0.2975	1		5	PCDH15	20	0.0057	0.147	0.9
3	BCL9L	2	0	0.2895	1		5	CDH13	19	0	0.1469	1
3	CDH12	2	0	0.2812	1		5	CDH22	19	0	0.1469	1
3	CDH8	2	0	0.2812	1		5	CDH3	19	0	0.1469	1
3	DACT1	2	0	0.3047	1		5	CELSR2	19	0	0.1469	1
3	TNRC6B	2	0	0.2991	1		5	CELSR3	19	0	0.1469	1
3	AJAP1	1	0	0.2809	0		5	DCHS1	19	0	0.1469	1
3	COPS3	1	0	0.2809	0		5	PCDH1	19	0	0.1469	1

3	GLIS2	1	0	0.2809	0		5	PCDH20	19	0	0.1469	1
3	HIC1	1	0	0.2809	0		5	PCDH7	19	0	0.1469	1
3	KANK1	1	0	0.2809	0		5	PCDHB11	19	0	0.1469	1
3	MGAT3	1	0	0.2809	0		5	PCDHB13	19	0	0.1469	1
3	MMP19	1	0	0.2809	0		5	PCDHB14	19	0	0.1469	1
3	PTPRU	1	0	0.2809	0		5	PCDHB16	19	0	0.1469	1
3	TFF2	1	0	0.2809	0		5	PCDHB18	19	0	0.1469	1
3	VEZT	1	0	0.2809	0		5	PCDHB3	19	0	0.1469	1
4	PTPN6	26	0.1112	0.3775	0.1169		5	PCDHB4	19	0	0.1469	1
4	SOCS1	21	0.0407	0.3164	0.3952		5	PCDHGA1	19	0	0.1469	1
4	SOCS3	21	0.0274	0.3061	0.3905		5	PCDHGC5	19	0	0.1469	1
4	TRIM63	13	0.0053	0.2426	0.8462		5	CDH12	1	0	0.1458	0
4	ASB1	12	0	0.2425	1		5	CDH8	1	0	0.1458	0
4	ASB10	12	0	0.2425	1		5	CDH9	1	0	0.1458	0
4	ASB11	12	0	0.2425	1		5	DCHS2	1	0	0.1282	0
4	ASB2	12	0	0.2425	1		6	RXRA	19	0.0357	0.136	0.0936
4	FBXO17	12	0	0.2425	1		6	PPARA	14	0.1137	0.1556	0.2418
4	FBXW8	12	0	0.2425	1		6	RXRG	12	0.0071	0.1354	0.1667
4	GAN	12	0	0.2425	1		6	CPT1B	6	0	0.1353	0.7333
4	SPSB4	12	0	0.2425	1		6	ACSBG1	4	0	0.1352	0.8333
4	UBE2S	12	0	0.2425	1		6	ACSL1	4	0	0.1352	0.8333
4	UBR2	12	0	0.2425	1		6	ACSL3	4	0	0.1352	0.8333
4	IFNA1	5	0.008	0.3145	0.4		6	FABP4	4	0	0.1199	0.5
4	IFNA14	5	0.008	0.3145	0.4		6	MED13L	4	0.0014	0.1352	0.8333
4	IFNA5	5	0.008	0.3145	0.4		6	MED25	4	0.0014	0.1352	0.8333
4	IL23R	4	0.0017	0.2777	0.5		6	APOA5	3	0	0.1352	0.6667
4	IL5RA	4	0.0017	0.2777	0.5		6	FABP1	3	0	0.1352	0.6667
4	IL9R	4	0.0017	0.2777	0.5		6	HMGCS2	3	0	0.1352	0.6667
4	KLRD1	3	0.0002	0.2847	0.6667		6	PLTP	3	0	0.1352	0.6667
4	SOCS5	3	0	0.2179	0		6	CYP27A1	2	0	0.1199	0
4	CD5	2	0	0.2845	1		6	OLR1	2	0	0.1199	0
4	DOK2	2	0	0.2701	1		6	ALDH1A2	1	0	0.1198	0
4	KLRC1	2	0	0.2744	1		6	DEGS2	1	0	0.1198	0

4	BTLA	1	0	0.2742	0		6	FAM120B	1	0	0.1198	0
4	CD33	1	0	0.2742	0		6	LIPA	1	0	0.1347	0
4	KIR3DL2	1	0	0.2742	0		6	SLC4A1	1	0	0.1198	0
4	ROS1	1	0	0.2742	0		6	WDTC1	1	0	0.1198	0
4	TRIM54	1	0	0.1954	0		7	CDC23	19	0.0176	0.1886	0.9006
5	RAC2	26	0.0816	0.3086	0.0738		7	UBE2C	19	0.0176	0.1886	0.9006
5	RAC3	23	0.0286	0.286	0.0909		7	UBOX5	19	0.0554	0.2006	0.8947
5	ARAP3	2	0	0.2361	1		7	ASB1	18	0	0.1715	1
5	ARHGAP12	2	0	0.2361	1		7	ASB10	18	0	0.1715	1
5	ARHGAP28	2	0	0.2361	1		7	ASB11	18	0	0.1715	1
5	ARHGAP29	2	0	0.2361	1		7	ASB18	18	0	0.1715	1
5	ARHGAP31	2	0	0.2361	1		7	ASB2	18	0	0.1715	1
5	ARHGAP32	2	0	0.2361	1		7	DET1	18	0	0.1715	1
5	ARHGAP33	2	0	0.2361	1		7	FBXO17	18	0	0.1715	1
5	ARHGAP40	2	0	0.2361	1		7	FBXO44	18	0	0.1715	1
5	ARHGAP9	2	0	0.2361	1		7	FBXW8	18	0	0.1715	1
5	ARHGEF12	2	0	0.2361	1		7	GAN	18	0	0.1715	1
5	DEPDC7	2	0	0.2361	1		7	RNF123	18	0	0.1715	1
5	DIRAS1	2	0	0.2361	1		7	SKP2	18	0	0.1715	1
5	GMIP	2	0	0.2361	1		7	SPSB4	18	0	0.1715	1
5	IQGAP3	2	0	0.2361	1		7	TRIM9	18	0	0.1715	1
5	MAP3K6	2	0	0.2361	1		7	UBE2S	18	0	0.1715	1
5	NCKAP1L	2	0	0.2361	1		7	UBR2	18	0	0.1715	1
5	OBSCN	2	0	0.2361	1		8	KCNAB1	11	0.0136	0.1456	0.2909
5	SRGAP1	2	0	0.2361	1		8	KCNAB3	11	0.0136	0.1456	0.2909
5	STARD13	2	0	0.2361	1		8	KCNA5	8	0.0717	0.1689	0.6429
5	STARD8	2	0	0.2361	1		8	KCNA3	7	0	0.1453	0.8571
5	PLAC8	1	0	0.236	0		8	KCNA6	7	0	0.1453	0.8571
6	LCK	22	0.0936	0.3627	0.1775		8	KCNB1	7	0.0022	0.1453	0.8095
6	CD3D	12	0.0133	0.3532	0.4545		8	KCNS3	7	0.0022	0.1453	0.8095
6	CD3G	12	0.0133	0.3532	0.4545		8	KCND3	6	0.0057	0.1275	0.4667
6	ACVR2B	10	0.0218	0.2379	0.1778		8	KCNF1	6	0.0022	0.1453	0.7333
6	ACVR1C	8	0.0113	0.2376	0.2857		8	KCNC3	2	0	0.1272	0

6	CD28	6	0.0034	0.3076	0.6		8	KCNC4	2	0	0.1272	0
6	PDCD1	6	0	0.297	0.9333		8	KCNH3	2	0	0.1272	0
6	CD274	5	0	0.2968	1		8	KCNH4	2	0	0.1272	0
6	PDCD1LG2	5	0	0.2968	1		8	KCNIP1	1	0	0.1131	0
6	CD86	4	0	0.2818	1		9	BMPR1B	13	0.0508	0.1753	0.0769
6	TDGF1	4	0.0275	0.2701	0.5		9	NOG	5	0.0056	0.1746	0.4
6	BMP6	3	0.0191	0.2584	0.3333		9	BMP6	3	0.0386	0.2095	0.3333
6	GDF1	3	0	0.2172	1		9	BMP7	3	0	0.1493	0.6667
6	AXL	2	0.0014	0.3069	0		9	FST	3	0	0.1493	0.6667
6	BMP3	2	0	0.1924	1		9	BMP8B	2	0	0.1493	1
6	BMP8B	2	0	0.1924	1		9	GDF15	2	0.0276	0.2093	0
6	GDF10	2	0	0.1924	1		9	GDF7	2	0	0.1493	1
6	GDF3	2	0	0.1924	1		9	INHBA	2	0	0.1493	1
6	EPHA7	1	0	0.2663	0		9	BMP3	1	0	0.1492	0
6	IGSF1	1	0	0.1923	0		9	CHRDL1	1	0	0.1492	0
6	SYNJ2BP	1	0	0.1923	0		9	GDF10	1	0	0.1492	0
6	TRAT1	1	0	0.2663	0		9	GDF3	1	0	0.1492	0
7	PLK1	12	0.0571	0.2856	0.2424		9	GREM1	1	0	0.1492	0
7	CDCA8	6	0	0.2229	1		10	USF1	15	0.0633	0.2327	0.0095
7	CLASP2	6	0	0.2229	1		10	ASPSCR1	1	0	0.1889	0
7	ESPL1	6	0	0.2229	1		10	CEL	1	0	0.1889	0
7	KIF2C	6	0	0.2229	1		10	CHI3L1	1	0	0.1889	0
7	PDS5B	6	0	0.2229	1		10	ENO3	1	0	0.1889	0
7	SPC24	6	0	0.2229	1		10	MYOC	1	0	0.1889	0
7	ALMS1	2	0	0.2224	1		10	PLAC8	1	0	0.1889	0
7	CEP72	2	0	0.2224	1		10	SERPINE2	1	0	0.1889	0
7	ASPM	1	0	0.2223	0		10	SLC19A1	1	0	0.1889	0
7	PBK	1	0	0.2223	0		10	SLC1A3	1	0	0.1889	0
7	TPPP3	1	0	0.2223	0		10	TFF2	1	0	0.1889	0
8	TJP1	10	0.0518	0.2852	0							
8	MPDZ	9	0.0003	0.1834	0							
8	CLDN11	2	0.0006	0.2223	0							
8	CLDN15	2	0.0006	0.2223	0							

8	CLDN19	2	0.0006	0.2223	0							
8	CLDN2	2	0.0006	0.2223	0							
8	CLDN22	2	0.0006	0.2223	0							
8	CLDN23	2	0.0006	0.2223	0							
8	CLDN4	2	0.0006	0.2223	0							
8	CLDN5	2	0.0006	0.2223	0							
8	CLDN8	2	0.0006	0.2223	0							
9	SCN1A	11	0.0465	0.301	0.4364							
9	NFASC	9	0.007	0.2331	0.6667							
9	NRCAM	8	0.0017	0.2329	0.8571							
9	SCN1B	8	0.0017	0.2329	0.8571							
9	SCN2B	8	0.0017	0.2329	0.8571							
9	ANK1	7	0.0017	0.2328	0.8571							
9	ANK3	7	0.0017	0.2328	0.8571							
9	SCN3A	6	0	0.1896	0.9333							
9	SCN7A	6	0	0.1896	0.9333							
9	CNTNAP1	1	0	0.1891	0							
10	DVL2	15	0.0863	0.3694	0.1619							
10	DVL1	11	0.0482	0.35	0.2545							
10	VANGL2	4	0	0.2705	0.8333							
10	PRICKLE1	3	0	0.2703	1							
10	PRICKLE2	3	0	0.2703	1							
10	DAAM1	2	0	0.2701	1							
10	DIXDC1	2	0	0.2701	1							
10	TRIM29	1	0	0.2699	0							
