# Supplementary material for: Focal exposure of limited lung volumes to high-dose irradiation down-regulated organ development-related functions and up-regulated the immune response in mouse pulmonary tissues
Source: BMC Genet. 2016 Jan 27;17:29. doi: 10.1186/s12863-016-0338-9 (PMC4729165; doi:10.1186/s12863-016-0338-9)
Supplement: Additional file 12: — Comparison of module composition between two network structures obtained from focally irradiated regions and non-irradiated neighboring lung regions. Both network structures were composed of 11 modules. Each spot represents a common gene present between modules. (PDF 272 kb) [file 12863_2016_338_MOESM12_ESM.pdf]

Additional file 12

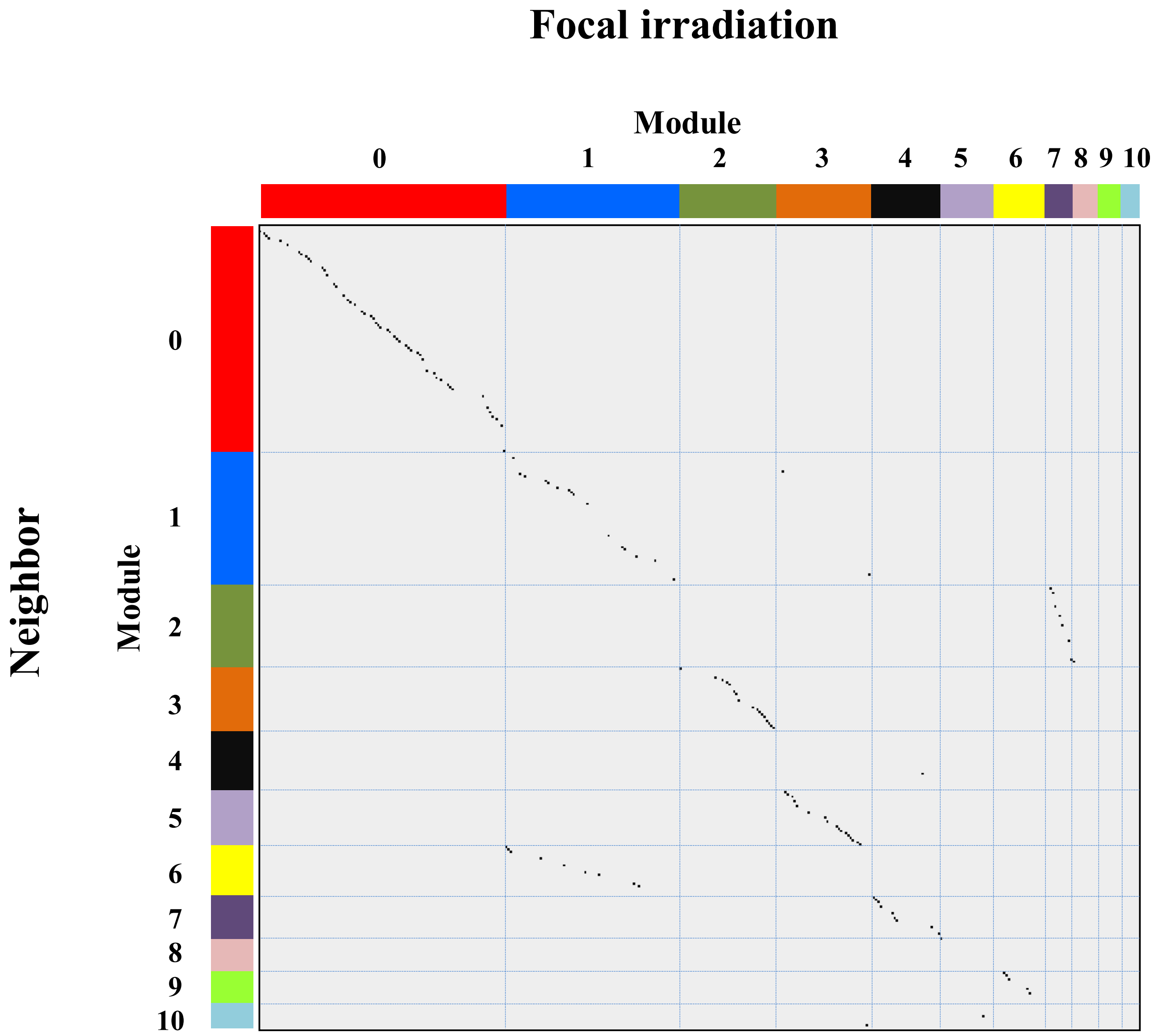

Additional file 12. Comparison of module composition between two network structures obtained from focally irradiated regions and non-irradiated neighboring lung regions. Both network structures were composed of 11 modules. Each spot represents a common gene present between modules.
