# Supplementary material for: Focal exposure of limited lung volumes to high-dose irradiation down-regulated organ development-related functions and up-regulated the immune response in mouse pulmonary tissues
Source: BMC Genet. 2016 Jan 27;17:29. doi: 10.1186/s12863-016-0338-9 (PMC4729165; doi:10.1186/s12863-016-0338-9)
Supplement: Additional file 13: — Differentially expressed genes between focally-irradiated regions and non-irradiated neighboring lung regions. Genes were selected using two classes times-series analysis implemented in Significance Analysis of Microarray (SAM) program [53]. Dotted lines represents the statistical significance level of FDR <0.01. Red circles represents over-expressed genes in focally-irradiated lung regions. (PDF 152 kb) [file 12863_2016_338_MOESM13_ESM.pdf]

Additional file 13

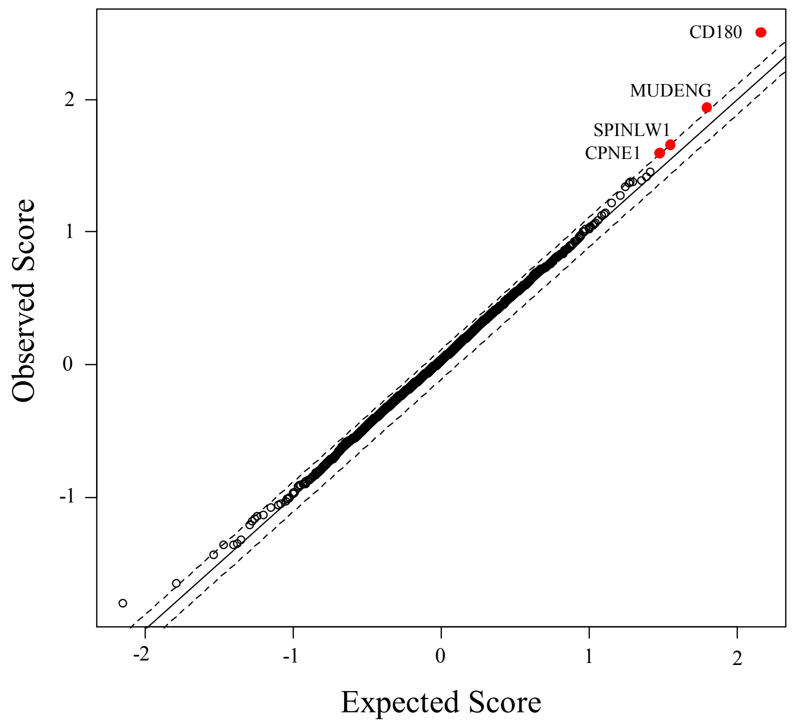

Additional file 13. Differentially expressed genes between focally-irradiated regions and non-irradiated neighboring lung regions. Genes were selected using two classes times-series analysis implemented in Significance Analysis of Microarray (SAM) program [Additional reference]. Dotted lines represents the statistical significance level of FDR<0.01. Red circles represents over-expressed genes in focally-irradiated lung regions.

[Additional reference]

Tusher VG, Tibshirani R, Chu G. Significance analysis of microarrays applied to the ionizing radiation response. Proc Natl Acad Sci U S A. 2001;98:5116-21.
